# Practical guide on left atrial appendage closure for the non-implanting physician: an international consensus paper

**DOI:** 10.1093/europace/euae035

**Published:** 2024-01-31

**Authors:** Tatjana Potpara, Marek Grygier, Karl Georg Häusler, Jens Erik Nielsen-Kudsk, Sergio Berti, Simonetta Genovesi, Eloi Marijon, Serge Boveda, Apostolos Tzikas, Giuseppe Boriani, Lucas V A Boersma, Claudio Tondo, Tom De Potter, Gregory Y H Lip, Renate B Schnabel, Rupert Bauersachs, Marco Senzolo, Carlo Basile, Stefano Bianchi, Pavel Osmancik, Boris Schmidt, Ulf Landmesser, Wolfram Doehner, Gerhard Hindricks, Jan Kovac, A John Camm

**Affiliations:** Medical Faculty, University of Belgrade, Belgrade, Serbia; University Clinical Centre of Serbia, Belgrade, Serbia; 1st Department of Cardiology, Poznan University School of Medical Sciences, Poznan, Poland; Department of Neurology, Universitätsklinikum Würzburg (UKW), Würzburg, Germany; Department of Cardiology, Aarhus University Hospital, Aarhus, Denmark; Ospedale del Cuore, Fondazione CNR Regione Toscana G. Monasterio, Pisa, Italy; School of Medicine and Surgery, University of Milano-Bicocca, Nephrology Clinic, Monza, Italy; Istituto Auxologico Italiano, IRCCS, Milan, Italy; Division of Cardiology, European Georges Pompidou Hospital, AP-HP, Paris, France; Cardiology, Heart Rhythm Management Department, Clinique Pasteur, Toulouse, France; Cardiologie Clinique Pasteur, Brussels University VUB, Brussels, Belgium; Ippokrateio Hospital of Thessaloniki, Aristotle University of Thessaloniki, Thessaloniki, Greece; Structural and Congenital Heart Disease, European Interbalkan Medical Centre, Thessaloniki, Greece; Cardiology Division, Department of Biomedical, Metabolic and Neural Sciences, University of Modena and Reggio Emilia, Policlinico di Modena, Modena, Italy; Cardiology Department, St. Antonius Hospital Nieuwegein/Amsterdam University Medical Centers, Amsterdam, Netherlands; Centro Cardiologico Monzino, IRCCS, Department of Clinical Electrophysiology & Cardiac Pacing, Department of Biomedical, Surgical and Dental Sciences, University of Milan, Milan, Italy; Cardiovascular Center Aalst, OLV Hospital, Aalst, Belgium; Liverpool Centre for Cardiovascular Science at University of Liverpool, Liverpool John Moores University and Liverpool Heart & Chest Hospital, Liverpool, UK; Danish Center for Health Services Research, Department of Clinical Medicine, Aalborg University, Aalborg, Denmark; Department of Cardiology, University Heart and Vascular Centre Hamburg, Hamburg, Germany; German Centre for Cardiovascular Research (DZHK), Partner Site Hamburg/Kiel/Lübeck, Hamburg, Germany; Cardioangiology Center Bethanien CCB, Frankfurt, Germany; Center for Vascular Research, Munich, Germany; Department of Surgery, Oncology and Gastroenterology, University Hospital of Padua, Padua, Italy; Division of Nephrology, Miull General Hospital, Acquaviva delle Fonti, Italy; EuDial Working Group of the European Renal Association, Acquaviva delle Fonti, Italy; Nephrology and Dialysis Unit, ASL Toscana NordOvest, Livorno, Italy; Department of Cardiology, University Hospital Kralovske Vinohrady, Charles University, Prague, Czech Republic; Cardioangiologisches Centrum Bethanien, Agaplesion Markus Krankenhaus, Frankfurt, Germany; Department of Cardiology, Angiology, and Intensive Care Medicine, Deutsches Herzzentrum Charité, Charité University Medicine, Berlin; Berlin Institute of Health-Center for Regenerative Therapies, Berlin, Germany; Deutsches Herzzentrum der Charité, Campus Virchow Klinikum, Berlin, Germany; German Centre for Cardiovascular Research (DZHK)- partner site Berlin, Charité Universitätsmedizin, Berlin, Germany; German Heart Center Charite, Campus Mitte, Berlin, Germany; Leicester NIHR BRU, University of Leicester, Glenfield Hospital, Leicester, UK; Genetic and Cardiovascular Sciences Institute, Cardiology Academic Group, St. George’s University of London, Cranmer Terrace, London SW190RE, UK

**Keywords:** Anticoagulation, Atrial fibrillation, Bleeding, Left atrial appendage closure, Left atrial appendage occlusion, Stroke, Prevention

## Abstract

A significant proportion of patients who suffer from atrial fibrillation (AF) and are in need of thromboembolic protection are not treated with oral anticoagulation or discontinue this treatment shortly after its initiation. This undertreatment has not improved sufficiently despite the availability of direct oral anticoagulants which are associated with less major bleeding than vitamin K antagonists. Multiple reasons account for this, including bleeding events or ischaemic strokes whilst on anticoagulation, a serious risk of bleeding events, poor treatment compliance despite best educational attempts, or aversion to drug therapy. An alternative interventional therapy, which is not associated with long-term bleeding and is as effective as vitamin K anticoagulation, was introduced over 20 years ago. Because of significant improvements in procedural safety over the years, left atrial appendage closure, predominantly achieved using a catheter-based, device implantation approach, is increasingly favoured for the prevention of thromboembolic events in patients who cannot achieve effective anticoagulation. This management strategy is well known to the interventional cardiologist/electrophysiologist but is not more widely appreciated within cardiology or internal medicine. This article introduces the devices and briefly explains the implantation technique. The indications and device follow-up are more comprehensively described. Almost all physicians who care for adult patients will have many with AF. This practical guide, written within guideline/guidance boundaries, is aimed at those non-implanting physicians who may need to refer patients for consideration of this new therapy, which is becoming increasingly popular.

## Graphical Abstract

**Graphical Abstract:**
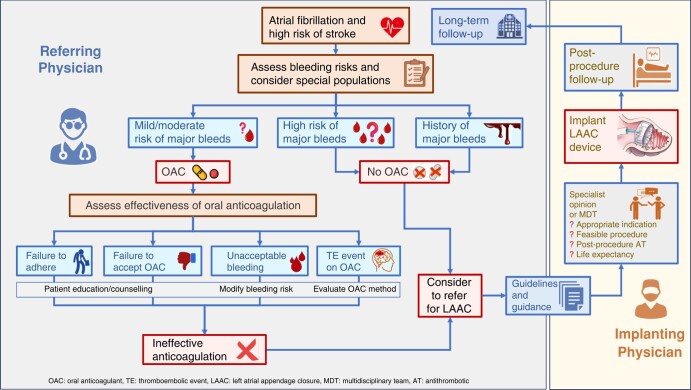

## Table of contents

Introduction
Evidence base for left atrial appendage closure
Indications for left atrial appendage closure
Referral considerations
 Responsibility of the referring physician
 Responsibility of the implanting physician
Current methods of percutaneous left atrial appendage closure
 Procedural steps
  Femoral venous puncture
  Transseptal access.
  Deployment of the occluder inside the left atrial appendage.
  Infective endocarditis prophylaxis
Left atrial appendage closure devices
Management of acute and early post-implantation complications
 Pericardial tamponade
 Device embolization
 Device-related thrombosis
 Procedure-related stroke
 Peri-device leak
Special populations
 Life-threatening or major gastrointestinal bleeding
 Cirrhosis and hepatic failure.
 Intracranial haemorrhage
 Ischaemic stroke in atrial fibrillation patients whilst on an oral anticoagulant
 Left atrial appendage thrombus despite optimal oral
 anticoagulant
 Coagulation disorders
  Important practical issues
  Indication for left atrial appendage closure implantation in haemostatic disorders
 Severely reduced glomerular filtration rate and kidney failure
 Prolonged dual antiplatelet therapy
 Left atrial appendage closure during/after other cardiac interventions.
  Left atrial ablation
  Left atrial appendage electrical isolation
  Transcatheter aortic valve replacement and left atrial
  appendage closure
  Transcatheter mitral valve edge-to-edge repair and left atrial appendage closure
  Left atrial appendage closure and other concomitant cardiac interventions (percutaneous coronary intervention, atrial
  septal defect, patent foramen ovale closures)
 Patient refusal/non-adherence/non-compliance
Anticoagulant/antiplatelet therapy regimens after left atrial
appendage closure
Post-discharge left atrial appendage closure patient follow-up
Other cardiac procedures after left atrial appendage closure
 Direct current cardioversion
 Atrial fibrillation catheter ablation
 Transcatheter mitral interventions, transcatheter aortic
 valve replacement, and percutaneous coronary intervention
Summary
Conclusions
Funding
References

## Acronyms and Abbreviations

ABC: atrial fibrillation better care
A_3_ICH: avoiding anticoagulation after intracerebral haemorrhage
ACP: Amplatzer cardiac plug
ACS: acute coronary syndrome
ACTIVE-A: atrial fibrillation clopidogrel trial with irbesartan for prevention of vascular events
ADALA: apixaban vs. dual antiplatelet therapy study after left atrial appendage occlusion
AFFIRMO: an integrated patient-centered holistic care pathway for the management of older patients with multi-morbidity to enhance cooperation among different health disciplines and promote a shared decision-making process
aMAZE: LAA ligation adjunctive to PVI for persistent or longstanding persistent atrial fibrillation
AMULET IDE: AMULET investigational device exemption
ANDES: short-term anticoagulation vs. antiplatelet therapy for preventing device thrombosis following left atrial appendage closure
APACHE-AF: apixaban after anticoagulation-associated intracerebral haemorrhage in patients with atrial fibrillation
APTT: activated partial thrombin clotting time
ARMYDA-AMULET: head-to-head comparison of single vs. dual antiplatelet treatment strategy after percutaneous left atrial appendage closure: a multi-centre, randomized study
AS: aortic stenosis
ASA: acetyl salicylic acid
ASAP-TOO: ASA Plavix feasibility study with WATCHMAN left atrial appendage closure technology
ASD: atrial septal defect
ASPIRE: anticoagulation in ICH survivors for stroke prevention and recovery
AVERROES: a phase III study of apixaban in patients with atrial fibrillation
AXADIA-AFNET: compare apixaban and vitamin-K antagonists in patients with atrial fibrillation and end-stage kidney disease
AZALEA-TIMI 71: safety and tolerability of abelacimab (MAA868) vs. rivaroxaban in patients with atrial fibrillation
BAFTA: Birmingham atrial fibrillation treatment of the aged study
BELIEF-RCT: effect of empirical left atrial appendage isolation on long-term procedure outcome in patients with persistent or longstanding persistent atrial fibrillation undergoing catheter ablation
CABG: coronary artery bypass grafting
CAP 2: continued access to PREVAIL
CAP 1: continued access to PROTECT
CATALYST: Amplatzer AMULET LAAO vs. NOAC
CHA_2_DS_2_-VASc: Congestive heart failure, Hypertension, Age >75 years, Diabetes mellitus, Stroke, Vascular disease, Age 65 -74 years, Sex Category (female)
CHAMPION-AF: WATCHMAN FLX vs. NOAC for embolic protection in the management of patients with non-valvular AF
CLEARANCE: comparison of left atrial appendage closure vs. oral anticoagulation in patients with non-valvular AF and status post-intracranial bleeding
CLOSURE-AF: left atrial appendage closure in patients with AF compared to medical therapy
COMBINE-AF: a collaboration between multiple institutions to better investigate non-vitamin K antagonist oral anticoagulant use in atrial fibrillation
COMPARE-LAAO: comparing effectiveness and safety of left atrial appendage occlusion to standard of care for atrial fibrillation patients at high stroke risk and ineligible to use oral anticoagulation therapy
CKD: chronic kidney disease
Cryo: cryotherapy
CT: computed tomography
CV: cardiovascular
CVA: cerebrovascular accident
DCCV: direct current cardioversion
DIC: disseminated intravascular coagulation
DOAC: direct oral anticoagulant
DRT: device-related thrombosis
ECG: electrocardiogram
eGFR: estimated glomerular filtration rate
ELAPSE: early closure of left atrial appendage for patients with atrial fibrillation and ischaemic stroke despite anticoagulation therapy
ENRICH-AF: edoxaban for intracranial hemorrhage survivors with atrial fibrillation
ESC: European Society of Cardiology
ESKD: end-stage kidney disease
EWOLUTION: registry on WATCHMAN outcomes in real-life utilization
FDA: Food and Drug Administration
GIB: gastro-intestinal bleeding
HAS-BLED: Hypertension, Abnormal renal/liver function, Stroke, Bleeding history or predisposition, Labile INR, Elderly (>65 years), Drugs/alcohol concomitantly
HD: haemodialysis
ICB: intracranial bleeding
ICE: intracardiac echocardiology
ICH: intracerebral haemorrhage
INR: international normalized ratio
INTERCEPT: carotid Implants for prevention of stroke recurrences from large vessel occlusion in atrial fibrillation patients treated with oral anticoagulation
ISTH: International Society on Thrombosis and Haemostasis
LAA: left atrial appendage
LAAC: left atrial appendage closure
LAA-KIDNEY: left atrial appendage closure in patients with non-valvular atrial fibrillation and end-stage chronic kidney disease
LAAO: left atrial appendage occlusion
LAAOS III/LAAOS-4: third/fourth left atrial appendage occlusion study
LAARGE: German left atrial appendage occlusion registry
LIBREXIA-AF: a study of milvexian vs. apixaban in participants with atrial fibrillation
LILAC-TIMI 76: study to evaluate the effIcacy and safety of abelacimab in high-risk patients with atrial fibrillation who have been deemed unsuitable for oral anticoagulation
LMWH: low molecular weight heparin
LPV: left pulmonary vein
LVEF: left ventricular ejection fraction
mAFA: mobile health (mHealth) technology for Improved screening and optimized Integrated care in atrial fibrillation
MDT: multidisciplinary team
MIRACLE-AF: a new model of integrated care of older patients with atrial fibrillation in rural China: a cluster randomization trial
NASPAF-ICH: non-VKA anticoagulants for stroke prevention in patients with AF and previous intracerebral haemorrhage
NCDR: National Cardiovascular Data Registry
NOAC: non-vitamin K oral anticoagulant
OAC: oral anticoagulant
OCEANIC-AF: a study to learn how well the study treatment asundexian works and how safe it is compared to apixaban to prevent stroke or systemic embolism in people with irregular and often rapid heartbeat (atrial fibrillation), and at risk for stroke
OCEANIC-AFINA: oral factor eleven a inhibitor asundexIan as novel antithrombotic-atrial fIbrillation untreated patients study
OCCLUSION-AF: left atrial appendage occlusion vs. novel oral anticoagulation for stroke prevention in AF: atrial fibrillation
OCEAN: optimal anticoagulation for higher risk patients post-catheter ablation for atrial fibrillation trial
OCEANIC-AF: a study to learn how well the study treatment asundexian works and how safe it is compared to apixaban to prevent stroke or systemic embolism in people with irregular and often rapid heartbeat (atrial fibrillation), and at risk for stroke
OPTION: comparison of anticoagulation with left atrial appendage closure after AF ablation
PCI: percutaneous coronary intervention
PDL: peri-device leak
PFO: patent foramen ovale
PINNACLE-FLX: protection against embolism for non-valvular AF subjects: investigational device evaluation of the WATCHMAN FLX LAA closure technology
PINNACLE: protection against embolism for non-valvular AF subjects
PRAGUE-17: left atrial appendage closure vs. novel anticoagulation agents in AF
PRESTIGE-AF: prevention of stroke in intracerebral haemorrhage survivors with atrial fibrillation
PREVAIL: evaluation of the WATCHMAN left atrial appendage closure device in patients with AF vs. long term warfarin therapy
PROTECT-AF: WATCHMAN left atrial appendage system for embolic protection in patients with AF
PT: prothrombin time
PVI: pulmonary vein isolation
RCT: randomized controlled trial
RENAL-AF: renal haemodialysis patients allocated apixaban vs. warfarin in atrial fibrillation
RENO-EXTEND: recurrent ischaemic stroke and bleeding in patients with atrial fibrillation who suffered an acute stroke while on treatment with nonvitamin K antagonist oral anticoagulants
RESTART: restart or stop antithrombotics randomized trial
RF: radiofrequency
SAFE LAAC CKD: optimal antiplatelet therapy following left atrial appendage closure in dialyzed patients
SE: systemic embolism
SoSTART: start or stop anticoagulants randomised trial
STABLED: stroke secondary prevention with catheter ablation and edoxaban for patients with non-valvular atrial fibrillation
STATICH: study of antithrombotic treatment after intracerebral haemorrhage
STOP-HARM: strategy to prevent hemorrhage associated with anticoagulation in renal disease management
STR-OAC: stroke despite OAC
STROKE-CLOSE: prevention of stroke by left atrial appendage closure in AF patients after intracerebral haemorrhage
SURPASS: surveillance post-approval analysis
SWISS-APERO: comparison of AMULET vs. WATCHMAN/FLX device in patients undergoing left atrial appendage closure
TAVI: transcatheter aortic valve implantation
TEER: transcatheter mitral valve edge-to-edge repair
TIA: transient ischaemic attack
TOE: transoesophageal echocardiogram
TTR: time in the therapeutic range
UFH: unfractionated heparin
USRDS: United States Renal Data System
VKA: vitamin K antagonist
VWF: von Willebrand factor
WASP: WATCHMAN Asia Pacific (registry)
WATCH-AF: watch bleeding episodes after left atrial appendage occlusion vs. usual care in patients with atrial fibrillation and severe to end-stage chronic kidney disease
WATCH-HD: left atrial appendage occlusion with WATCHMAN device in patients with non-valvular atrial fibrillation and end-stage chronic kidney disease on haemodialysis
WM/WM-FLX: WATCHMAN/WATCHMAN FLX.

## Introduction

Atrial fibrillation (AF) is the most common sustained cardiac arrhythmia in adults and is associated with increased morbidity and mortality, mainly caused by embolic strokes and the development of heart failure.^1^ Due to longer life expectancy and better treatment of conditions associated with high AF risk, such as heart failure, the prevalence and incidence of AF have been continuously rising.^2^

There are multiple anticoagulant drugs, predominantly from two classes: vitamin K antagonists (VKAs), which reduce the synthesis of functional coagulation factors and direct oral anticoagulants (DOACs), which inhibit the action of certain coagulation factors. Since these agents increase the risk of bleeding, doctors, patients and caregivers are sometimes reluctant to use them.

Oral anticoagulation (OAC) is highly effective in preventing cardioembolic strokes in AF patients. In the trials comparing VKAs with placebo, OAC reduced the risk of stroke by 64% and all-cause mortality by 26%.^3^ However, in Europe and North America, VKAs have been almost entirely replaced by DOACs in the management of non-valvular AF patients. These drugs are comparable to VKAs in preventing ischaemic stroke, but superior in terms of bleeding risk. In a meta-analysis of trials comparing VKA with DOACs, with more than 70 000 patients with AF, treatment with DOACs was associated with a significant reduction in all strokes by 19%, which was mainly driven by a significant reduction in haemorrhagic stroke (HR 0.49, 95% CI 0.38–0.64).^4^ However, there remains a residual risk of stroke 0.8 per 100 patient-years.^5^

Notwithstanding the impressive reduction in the risk of intracerebral bleeding with DOACs, the risk of major bleeding in the gastrointestinal tract is not much reduced in comparison to VKAs, and may actually be increased as compared to VKAs with some DOACs.^4^ However, DOACs do not inhibit coagulation Factor VII which is fundamentally important for haemostasis but not so relevant for thrombosis.^6^ Although the balance between stroke prevention and major bleeding is improved with DOACs, the bleeding problem is not eliminated.^7^ The major bleeding rate remains between 1 and 3 per 100 patient-years, but over a 3-year period, it was 11% in the LAAC/OAC meta-analysis and in the DOAC vs. VKA pre-approval trials, it was 5.9% with DOACs over 32 months.^8^ In AF patients with a GI bleed whilst taking anticoagulant, there is a very high risk of a recurrent bleed (27 per 100 patient-years).^9^

In patients who have suffered serious bleeding and/or are at high risk of bleeding or in whom VKA/DOAC treatment has failed to prevent AF-related stroke, an interventional technique may be considered. The use of non-pharmacological thromboprophylaxis would also significantly reduce the long-term anticoagulant drug burden. Amongst these techniques, closure or occlusion of the left atrial (LA) appendage,^10^ the intracardiac site at which most thrombi form in patients with AF, can be achieved by a reasonably safe catheter-based procedure known as LA appendage closure (LAAC) or LA appendage occlusion (LAAO).

This procedure is being increasingly offered in developed countries as a robust alternative to OAC for those in need, but the knowledge of LAAC is often modest outside the interventional cardiology and electrophysiology communities. On the other hand, the patients who might benefit from this therapeutic approach are often under the care of a general cardiologist, general or primary care physician, gerontologist, nephrologist, gastroenterologist, neurologist or stroke physician, etc. An understanding and appreciation of the value and applicability of LAAC are needed by all of those who care for patients with AF at risk of stroke but with a medical history, comorbidity, or lifestyle that prevents adequate anticoagulation.

This practical guide, written by an international multidisciplinary group consisting of members of the European Society of Cardiology, the ESC Stroke Council and cardiologists and physicians from other interested specialties, aims to provide an overview of the principles, patient selection, follow-up, and limitations of LAAC. The scope is to provide practical information about LAAC to the general medical community dealing with such AF patients, and not a manual for those who implant the device.

## Evidence base for left atrial appendage closure

The efficacy and safety of LAAC were first shown in the randomized PROTECT-AF (data collection from 2005) and PREVAIL (data collection from 2010) clinical trials in which AF patients without obvious contraindications to warfarin were randomized to either LAAC with WATCHMAN (with warfarin and aspirin for at least 45 days after the procedure) or warfarin aiming at an INR of 2–3 (*n* = 1114). After a 5-year follow-up, LAAC provided stroke prevention comparable to VKA with a significant reduction in major bleeding, haemorrhagic stroke, disabling/fatal stroke, cardiovascular (CV) death, and all-cause death.^11^

The PRAGUE-17 randomized trial (data collection from 2015) compared LAAC (AMULET or WATCHMAN) with DOAC, mainly apixaban, (*n* = 402) showing non-inferiority for LAAC in the prevention of stroke/transient ischaemic attack (TIA), CV death, clinically relevant bleeding, and superiority in preventing non-procedural bleeding over 4 years.^12^

**Figure 1:**
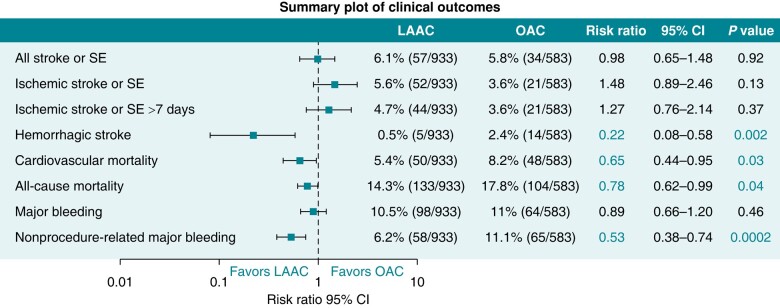
Clinical outcomes from the PROTECT, PREVAIL and PRAGUE-17 randomized clinical trials. Adapted with permission from Turagam et al.^13^ LAAC, left atrial appendage closure; OAC, oral anticoagulation; SE, systemic embolism.

*Figure 1* shows clinical outcomes from the three RCTs comparing LAAC vs. VKA/DOAC.^13^ It can be seen that the point estimate for the ischaemic stroke rate is 5.6% with LAAC compared with 3.6% with OAC. This adverse trend is not significant but is a concern that detracts from a more fulsome acceptance of LAAC therapy as a legitimate alternative to OAC prophylaxis. However, a propensity-matched analysis has suggested that strokes in patients with LAAC are less disabling than those seen in patients receiving DOAC therapy.^14^

There are multiple observational studies and registries of AF patients undergoing LAAC with various devices (Amulet Cardiac Plug, ACP; AMULET; Watchman; WATCHMAN FLX) mostly showing a 60–80% reduction in the rate of ischaemic stroke and major bleeding compared with predicted rates based on the CHA_2_DS_2_-VASc and HAS-BLED score values (e.g. ACP registry,^15^ AMULET Observational Study,^16^ EWOLUTION,^17^ NCDR-LAAO registry,^18,19^ PINNACLE-FLX^20^).

A recent meta-analysis of studies comparing LAAC to DOAC (*n* = 4411) showed the risk of stroke/TIA to be similar with LAAC and DOAC, whereas LAAC was superior in reducing CV mortality, major and non-major bleeding.^21^ In the randomized LAAOS-III study (*n* = 4770), surgical LAAC in addition to DOAC (continued in about 70% of all study patients) was associated with a 33% reduction in the risk of stroke/TIA after 3 years.^22^ Factor XI inhibitors are currently being investigated for thromboprophylaxis in AF patients with a high risk of thromboembolic events. Ongoing trials include OCEANIC-AF and OCEANIC-AFINA with asundexian,^23^ AZALEA-TIMI 71,^24^ LILAC-TIMI 76 with abelacimab,^25^ and LIBREXIA-AF with milvexian and compare Factor XI inhibitors against DOACs or placebo.^26^ If these new drugs can prevent thromboembolism without a substantial bleeding risk, a comparison with LAAC will be needed. However, OCEANIC-AF has been terminated prematurely for lack of asundexian efficacy when compared with apixaban. On the other hand, the AZALEA trial was also terminated prematurely but because there was substantially less bleeding with abelacimab than with rivaroxaban. Even if Factor XI inhibitors are not as effective as DOACs but more effective than placebo with a substantial reduction in bleeding when compared with conventional anticoagulation there might still be an important role for these agents in patients who cannot use standard agents.

Currently, there is no randomized controlled trial (RCT)-based data on LAAC in patients who are intolerant of or contraindicated for OAC. Data on such patients is very much needed because this is actually the subgroup of AF patients that is treated with LAAC in clinical practice today and the subgroup that would likely have the greatest benefit from LAAC (*Table 1*). However, patient recruitment has been slow into these trials, e.g. ASAP-TOO,^27^ CLOSURE-AF,^28^ STROKE-CLOSE,^29^ CLEARANCE,^30^ COMPARE-LAAO,^31,32^ and LAA-KIDNEY^33^ amongst others. The ASAP-TOO trial was terminated prematurely due to slow enrolment but patient follow-up is still active.

**Table 1:** Ongoing randomized trials comparing LAAC vs. best medical care in AF patients with contraindications for long-term anticoagulation

	CLOSURE-AF^28^	STROKE-CLOSE^29^	CLEARANCE^30^	LAA-KIDNEY^33^	COMPARE-LAAO^31,32^
Patient population	AF and high bleeding risk (HAS-BLED ≥3; prior major bleeding; CRF)	AF and ICH within 12 months	AF and ICH or intracerebral amyloid vasculopathy	AF and end-stage kidney disease	NVAF pts with CHA₂DS₂-VASc ≥ 2 and absolute contraindication to (D)OAC
Number of patients	1000	600	530	430	609
Randomization	LAAC vs. best medical care	AMULET vs. best medical care (2:1)	WATCHMAN FLX vs. best medical care	AMULET vs. best medical care	AMULET or WATCHMAN FLX vs. nothing ± APT (2:1)
Primary endpoint	Stroke, SE, major bleeding or CV death at 2 years	Stroke, SE, major bleeding or all-cause mortality at 2 years	Stroke, SE, major bleeding or CV death at 3 years	Time to first stroke, SE, CV death or major bleeding	1. Any stroke. 2. Composite of stroke, TIA, and SE

APT, antiplatelet therapy; CV, cardiovascular; CHA_2_DS_2_-VASc, congestive heart failure, hypertension, age ≥75 years, diabetes mellitus, stroke, vascular disease, age 65–74 years, sex category (female); ICH, intracerebral bleeding; LAAC, left atrial appendage closure; SE, systemic embolism; TIA, transient ischaemic attack.

Based on the currently available evidence and clinical experience, LAAC is now being investigated in broad populations of AF patients in large-scale trials. In the OPTION trial,^34,35^ AF patients undergoing catheter ablation for AF were randomized to LAAC or DOAC after ablation. In the CHAMPION-AF trial^36^ and CATALYST trial,^37^ AF patients with no contraindications to DOACs and CHA_2_DS_2_-VASc of ≥2 for men and CHA_2_DS_2_-VASc of ≥3 for women are randomized to LAAC or DOAC (*Table 2*). In the OCCLUSION-AF trial,^38^ AF patients with a recent ischaemic stroke are randomized to either LAAC or DOAC.^39^

**Table 2:** Ongoing large-scale randomized trials comparing LAAC vs. DOAC

	OPTION^35^	CHAMPION-AF^36^	CATALYST^37^
Patient population	CHA_2_DS_2_-VASc ≥ 2 (men) CHA_2_DS_2_-VASc ≥ 3 (women)	CHA_2_DS_2_-VASc ≥2 (men) CHA_2_DS_2_-VASc ≥ 3 (women)	CHA_2_DS_2_-VASc ≥ 3 initially, now updated to CHA_2_DS_2_-VASc ≥ 2 (men) CHA_2_DS_2_-VASc ≥ 3 (women)
Number of patients	1600	3000	2650
Randomization	WM-FLX vs. OAC	WM-FLX vs. DOAC	AMULET vs. DOAC
Primary endpoint	Stroke, SE, or death at 3 years (non-inferiority) Major or clinically relevant bleeding at 3 years (superiority)	Stroke, SE, or CV death at 3 years (non-inferiority) Major or clinically relevant bleeding at 3 years (superiority)	Stroke, SE, or CV at 2 years (non-inferiority) Major or clinically relevant bleeding at 2 years (superiority)
Enrolment status	Completed	Completed	Enrolling

CV, cardiovascular; CHA_2_DS_2_-VASc, congestive heart failure, hypertension, age ≥75 years, diabetes mellitus, stroke, vascular disease, age 65–74 years, sex category (female); DOAC, direct oral anticoagulant; WM-FLX, WATCHMAN FLX; SE, systemic embolus.

There are also several observational studies on special AF patient sub-populations undergoing LAAC (i.e. patients with prior ICH, prior ischaemic stroke, renal failure, stroke despite anticoagulation) suggesting a net benefit of LAAC in the prevention of stroke and bleeding. Some of those studies are propensity score-matched comparing LAAC in AF patients with a prior ICH to standard therapy^40^ or LAAC to DOAC.^41^

## Indications for left atrial appendage closure

Stroke reduction in patients with AF requires more than thromboprophylaxis, hence the move toward a holistic or integrated care approach to AF management. This is recommended in guidelines as the Atrial fibrillation Better Care pathway.^42^ Adherence with this evidence-based strategy is associated with a 31% reduction in stroke, as well as lower mortality and bleeding, and is supported by various retrospective and prospective cohort studies from different parts of the world,^43^  *post hoc* analysis from adjudicated outcomes from clinical trials.^44,45^

Transcatheter LAAC has been increasingly used as an anti-thrombotic approach in patients with AF, especially in the United States of America.^18,46^ While contemporary European AF registry-based studies reported a <1% use of LAAC in clinical practice,^47,48^ a trend toward increasing use of LAAC in Europe has been recently observed, including the changing profile of AF patients undergoing the procedure (i.e. less frail and generally less comorbid patients).^49^

**Figure 2:**
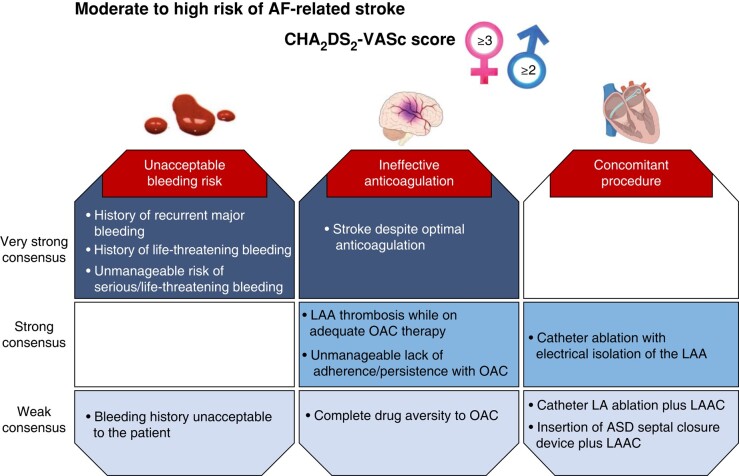
Possible candidates for LAAC. ASD, atrial septal defect; CHA_2_DS_2_-VASc, congestive heart failure, hypertension, age ≥75years, diabetes mellitus, stroke, vascular disease, age 65–74 years, sex category (female); LAA, left atrial appendage; LAAC, left atrial appendage closure; OAC, oral anticoagulation.

Guideline recommendations and consensus statements considering the use of transcatheter LAAC for the prevention of stroke and systemic thromboembolism in patients with AF are summarized in *Tables 3*, *4* and *Figure 2*.

**Table 3:**
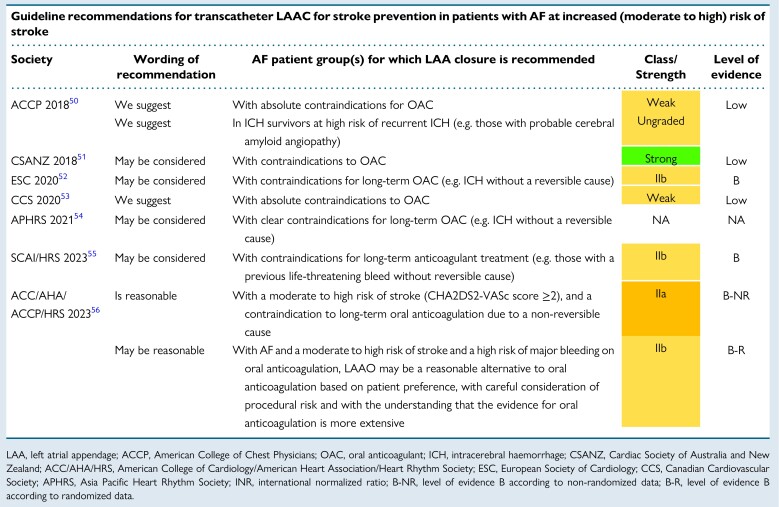
Recommendations for the use of LAA closure in the international guideline documents

**Table 4:**
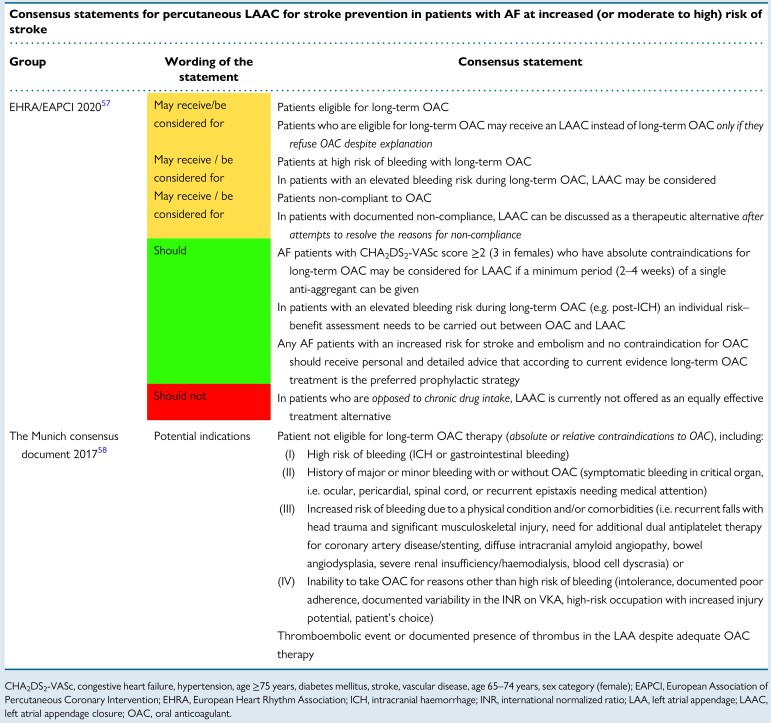
Recommendations for the use of LAA closure in consensus statements

Formal guideline documents have consistently recommended percutaneous LAAC for AF patients with contraindications to long-term OAC, using a low class of recommendation and low level of evidence, although the 2023 ACC/AHA/ACCP/HRS guidelines have recently upgraded this to a Level IIa recommendation and have added a IIb recommendation for LAAO as an alternative to OAC in patients with a high stroke and bleeding risk (*Table 3*).^50–56,59^ Consensus documents explain the recommendations in more detail and extend the implications (*Table 4*),^57,58^ thus also including AF patients who:

- suffer major bleeding events during anticoagulant therapy
- have a high risk of non-modifiable anticoagulant bleeding
- had a thromboembolic event or LAA thrombosis whilst on optimal OAC^60^
- refuse or are non-compliant to long-term OAC
- undergo catheter ablation with electrical isolation of the LAA

Both guideline and consensus documents/position papers aim to inform clinical practice. Methodological differences (rigid interpretation of the evidence base, particularly clinical trials for guidelines, and a less formal process also utilizing observational data for consensus documents) result in official professional society recommendations in guidelines and broader non-official advice, in consensus documents.^61^

The most recent consensus documents addressing the use of transcatheter LAAC for the prevention of stroke and systemic embolism (SE) in patients with AF emphasize that LAAC should not be *routinely* offered to patients unwilling to take OAC therapy or who are simply non-compliant with their anticoagulation medication, before providing them with detailed counselling. Careful individual risk–benefit assessment and shared decision-making should be undertaken in each patient^62^. The common indications for LAAC are listed in Box 1.

**Box 1:** When to consider referral for LAAC

AF and significant risk of stroke CHA_2_DS_2_-VASc ≥2 (men) CHA_2_DS_2_-VASc ≥3 (women) and:
History of recurrent or irremediable major bleeding
Recurrent non-major bleeding
Predicted high risk of bleeding (HAS-BLED ≥3)
Bleeding disorder (coagulopathy or angiodysplasia)
Indication for long-term antiplatelet therapy
Cerebral microbleeds/amyloid cerebral vasculopathy
Advanced renal failure including dialysis
Hepatic failure
Stroke despite appropriate OAC
Non-adherence to OAC despite attempts to educate the patient
Electrically isolated LAA after ablation

## Referral considerations

### Responsibility of the referring physician

All patients with AF who are being considered for any cardiac intervention must be assessed by taking a cardiac history relating to the presence of AF, major structural or functional heart disease, potentially reversible causes of bleeding, or alternative causes of stroke besides AF. Routine investigations including 12-lead surface electrocardiogram and basic laboratory tests will have been performed before a patient is considered for LAAC therapy.

The need for thromboembolic protection in patients with AF must be firmly established utilizing risk scores such as CHA_2_DS_2_-VASc. Their bleeding risk should also be assessed using a validated structured bleeding risk assessment that addresses modifiable and non-modifiable bleeding risks, such as the HAS-BLED score. Any additional factor leading to an increased thromboembolic or bleeding risk should also be documented.

### Responsibility of the implanting physician

The first responsibility of the interventional specialist is to confirm the indication for LAAC. There is a practical value of holding a multidisciplinary team (MDT) meeting to assess patients who have been or are to be referred for LAAC. As the indication is often for non-cardiac problems (neurological, gastrointestinal, haematological, renal, etc.) such an MDT can assess patient data at an early stage and achieve consensus on the management plan.

In some healthcare systems (e.g. National Institute for Health and Care Excellence), an ‘MDT’ is mandatory for selecting patients for LAAC since it helps reduce selection bias, streamlines referrals and facilitates optimal patient management.^63^

Pre-procedural diagnostic workup usually includes transoesophageal echocardiography (TOE) or cardiac computed tomography (CT) to delineate LAA anatomy and suitability for closure, and to rule out LAA thrombosis. Left atrial appendage thrombosis can also be excluded using TOE or intracardiac echocardiography (ICE) at the beginning of the procedure.^64^ In general, the presence of LAA thrombus is considered as a contraindication to LAAC. Nonetheless, several case series of LAAC have been reported in patients with a thrombus present only in the distal part of the LAA^65^—see below.

The selection of a specific LAA closure device and its size will depend on the operator's experience and the LAA anatomy as assessed by pre-procedural CT or TOE and by peri-procedural TOE or ICE and selective LAA angiography. Cardiac CT offers a better understanding of LAA anatomy and the most accurate measurements.^66,67^ There are several dedicated software packages for planning a LAAC procedure based on cardiac CT.

If the patient is on a DOAC, the treatment may be stopped one day before the procedure (i.e. last dose of rivaroxaban or edoxaban in the morning, or apixaban and dabigatran in the evening before the procedure) without bridging. Box 2 shows the range of investigations and preparations undertaken by the implanting centre prior to LAAC.

**Box 2:** Before LAAC at implanting center

Clinical examination and biochemistry: rule out infection; assess renal function
TTE: LV function, valves, pericardium
Cardiac CT or TOE: LAA anatomy; device selection and size; rule out LAA thrombus
Stop OAC; loading dose of antiplatelets
Intravenous prophylactic antibiotics

## Current methods of percutaneous left atrial appendage closure

### Procedural steps

Left atrial appendage closure is a standardized procedure that requires specific training of the implanter and interventional team. It is most often undertaken under general anaesthesia and is guided by TOE, but ICE or micro-/mini-TOE is increasingly used making it possible to perform the procedure with local analgesia and light sedation.

#### Femoral venous puncture

Femoral venous access is usually obtained under ultrasound guidance to reduce the risks of vascular complications.^68–72^

#### Transseptal access

Transseptal puncture is a crucial step to safely access the left atrium and successfully deploy a LAAC device. This technique requires specific training and has a learning curve.

#### Deployment of the occluder inside the left atrial appendage

**Figure 3:**
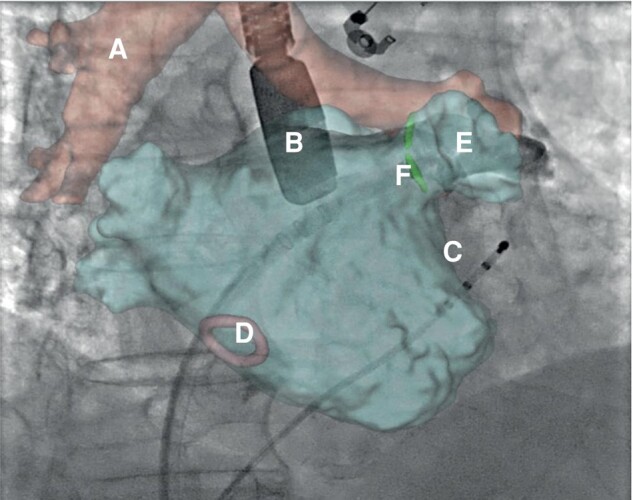
Fluoroscopy image with a 3D reconstructed CT-scan image fusion in order to guide LAA occluder positioning and deployment. A: tracheal landmark used for the fusion between the CT-scan image and the fluoroscopy system; B: transoesophageal echocardiography probe used to guide the LAA occluder positioning; C: quadripolar catheter placed inside the coronary sinus in order to guide the transseptal puncture (optional); D: transseptal puncture area; E: left atrial appendage (LAA) in right anterior projection; F: catheter positioned in front of the LAA entrance before occluder release.

Procedural imaging is of crucial importance for a successful LAAC. The procedure is guided by TOE or ICE, depending on the operator's experience. Device deployment is additionally controlled by fluoroscopy and fusion of pre-procedural CT images with fluoroscopy is occasionally used (*Figure 3*). Transoesophageal echocardiogram/intracardiac echo is also crucial to confirm the optimal placement of the device and complete sealing of the LAA.

#### Infective endocarditis prophylaxis

Peri-procedural antibiotic prophylaxis and surgical standard aseptic measures in the catheter laboratory environment are recommended during the LAA implant procedure (ESC guidelines). Elimination of potential sources of sepsis (including of dental origin) should be considered 2 or more weeks before implantation.^73^

### Left atrial appendage closure devices

**Figure 4:**
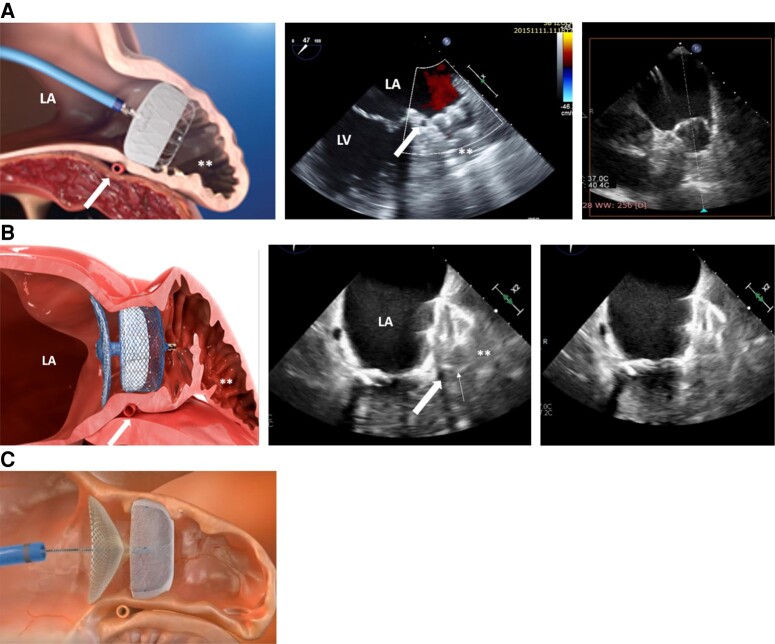
(*A*) WATCHMAN FLX (Boston Scientific). The WATCHMAN FLX is deployed at the proximal part of the LAA, at the level of the circumflex artery and the ridge. There are two rows of anchors distributed across the distal half of the device. Small arrow: circumflex artery; large arrow WATCHMAN FLX; **, distal part of the LAA; LA, left atria; LV, left ventricle. (*B*) AMULET (Abbott). The AMULET is deployed at the proximal part of the LAA, at the level of the circumflex artery, and the ridge. AMULET is a dual-seal technology consisting of a lobe to anchor in the neck of the LAA and a disc to close off the opening into the LAA. Small arrow: circumflex artery; large arrow: the lobe of the AMULET; **, distal part of the LAA; LA, left atrium. (*C*) LAmbre (Lifetech) offers a design very similar to the Amulet, with a distal anchoring umbrella and a proximal disc.

**Figure 5:**
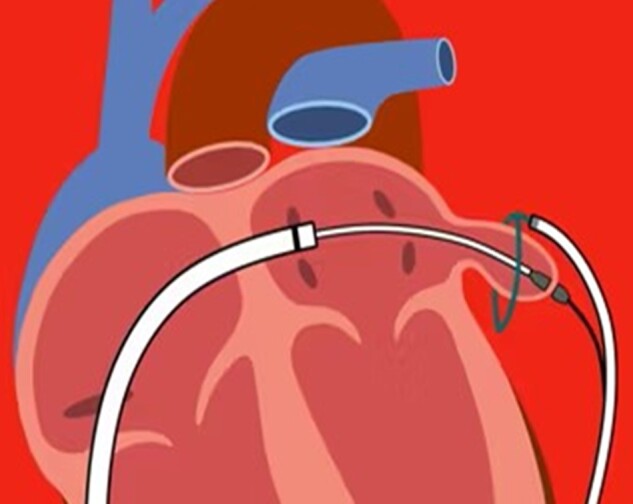
Lariat suture delivery device (SentreHeart). After proper alignment, the Lariat suture is tightened from the epicardium, providing a ligature of the LAA at its neck.

A range of devices has been developed in order to provide safe and efficient LAAC (*Table 5*).^74–79^ Of these, the WATCHMAN FLX, AMULET, and LAmbre devices have been extensively studied (*Figure 4A–C*). Another form of LA occlusion may be achieved using a noose inserted epicardially around the os of the LAAC—the LARIAT device (*Table 5* and *Figure 5*).

**Table 5:** Different types of occluders currently in use and their characteristics

	Company	Structure	Features	Limitations
WATCHMAN FLX (*Figure 4A*)^74–76^	Boston Scientific, Marlborough, MA, USA	Endocardial Single component	High degree of conformability, sealing and safety	Shallow LAAs with proximal bifurcation
AMPLATZER AMULET-ACP (*Figure 4B*)^77^	Abbott, St Paul, MN, USA	Endocardial Dual component	Possible to seal complex LAA anatomies	More complex to manoeuvre
LAmbre (*Figure 4C*)^78^	Lifetech Scientific, Shenzhen, China	Endocardial Dual component	Possible to seal complex LAA anatomies	More complex to manoeuvre
LARIAT (*Figure 5*)^79^	SentreHeart, Redwood City, CA, USA	Epicardial suture	Adjustable size No need for post-procedural OAC	Both epicardial and endocardial access Post-procedural pericardial pain Not suitable when prior cardiac surgery or thoracic radiation

LAA, left atrial appendage; OAC, oral anticoagulant.

Since the LAAC technique is becoming increasingly popular, many other devices are under development.

## Management of acute and early post-implantation complications

Left atrial appendage closure has become a relatively low-risk procedure (*Table 6*).^80–83^ Peri-procedural complications are listed in *Table 6* and benefits and risks are set out in Box 3. Some complications may occur over the longer term, such as late pericardial effusions or device-related thrombosis (DRT) and all physicians following patients post-procedure must be aware of these. Complications occur more commonly in patients with a higher CHA_2_DS_2_-VASc score.^84^

**Table 6:** Incidence of peri-procedural LAAC complications

Complication	SURPASS registry	AMULET IDE
Pericardial tamponade/effusion	0.32%	2.4%
Device embolization	0.01%	0.7%
Stroke	0.09%	0%
Death	0.07%	0%
Device-related thrombosis at 45 days	0.23%	2.2%
Peri-device leaks at 45 days	12.9% (<3 mm) 3.7% (3–5 mm) 0.4% (>5 mm)	27% (<3 mm) 9% (3–5 mm) 1% (>5 mm)

Data were derived from the SURPASS registry of 66.894 WATCHMAN FLX implants performed in the US from August 2020 to March 2022 and from 915 AMULET implants in the randomized AMULET IDE trial 2016–2020.^81–83^

### Pericardial tamponade

Pericardial effusion or tamponade represents a serious complication. Its incidence has decreased from the initially reported rate of 4.3% in the PROTECT-AF trial,^85^ to 0.3% in the SURPASS study that included 16 048 WATCHMAN FLX implants.^81^

Most tamponades/effusions occur during the procedure or within 24 h. To minimize their occurrence, imaging guidance with TOE/ICE is essential for all procedural phases, from a transseptal puncture to device placement and release.

LAA perforation can sometimes be managed just by finalizing the LAA device implantation. For significant active pericardial bleeding, auto-transfusion is possible to minimize blood loss and the need for transfusion. Reversal of anticoagulation should be considered only in cases with severe haemodynamic deterioration. Surgical intervention is rarely needed (*Table 7*).

**Table 7:** Mechanisms of pericardial effusion and tamponade and their prevention and treatment

Most frequent mechanisms of pericardial effusion/tamponade
Transseptal puncture
Manipulation of a stiff guidewire
Recurrent repositioning of the device
Deep positioning of the device
**How to prevent effusion/tamponade?**
CT scan/TOE pre-procedure
TOE/ICE intra-procedure
Angio intra-procedure
**Pericardial effusion/tamponade—what to do?**
Percutaneous drainage in the catheter laboratory
Blood transfusion
Intensive care unit
Surgical drainage as backup

The table lists the most frequent mechanisms of pericardial effusion and actions to prevent and to manage them.

ICE, intracardiac echocardiography; TOE, transoesophageal echocardiogram; CT, computed tomography.

Although most pericardial effusions occur within 24 h of LAAC, late pericardial effusions can rarely occur. If a pericardial effusion is suspected, the patient should be immediately referred to the implanting centre or the nearest cardiology site for echocardiography and possible pericardiocentesis.

While acute pericardial effusion/tamponade is related to trauma to the left atrium, pulmonary veins, or the LAA that may occur during the procedure, it is often difficult to identify the mechanism of late effusions and other common causes of pericardial effusion should also be considered.

### Device embolization

**Figure 6:**
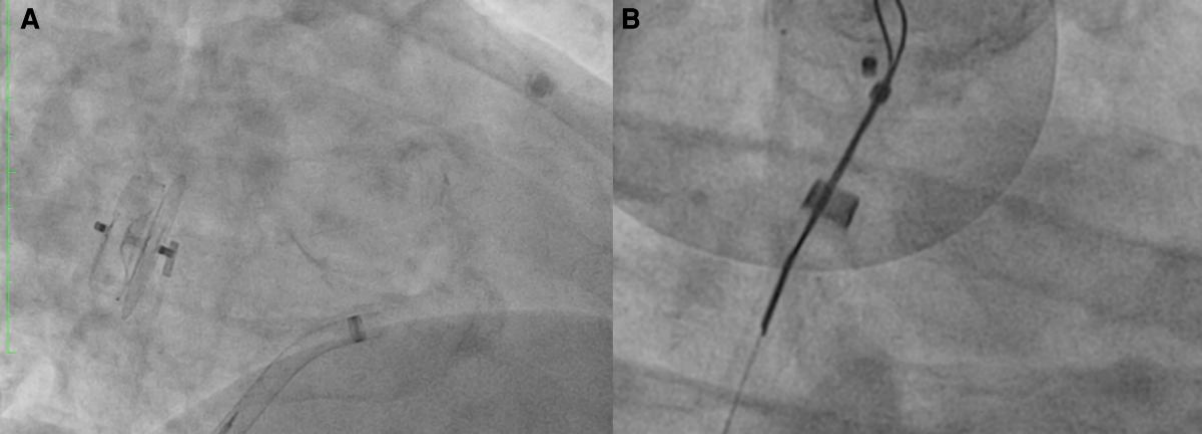
Embolization of an ACP device (Abbott) to the LA due to inappropriate sizing (*A*). Effective device retrieval with a goose neck snare (*B*).

Device embolization has become a rare complication with the most recent LAAC devices (0.01% with WATCHMAN FLX in SURPASS). The risk of embolization is increased with device under-sizing, very proximal implantation, misalignment of the device to the axis of the LAA, and sinus rhythm (*Table 8*). Device embolization can to a large extent be prevented by adequate pre-procedural and intra-procedural imaging. Smaller LAAC devices that embolize will most often travel through the left heart and aortic valve to the descending aorta, whereas larger devices will remain in the LA or LV. Device embolization is rarely associated with haemodynamic deterioration. Percutaneous retrieval is usually successful with a snare catheter or retrieval forceps (*Figure 6*). If the device becomes entangled in the mitral valve apparatus, percutaneous snaring can potentially damage the valve and acute surgery might be required.

**Table 8:** Mechanisms of device embolization and its treatment

Most frequent mechanism of device embolization
Device under-sizing
Too proximal implantation of the device
Inadequate coaxial placement of the device within LAA
Sinus rhythm
**Device embolization—what to do?**
Catheter-based retrieval of devices
Surgical removal of the device (rarely needed)

### Device-related thrombosis

**Figure 7:**
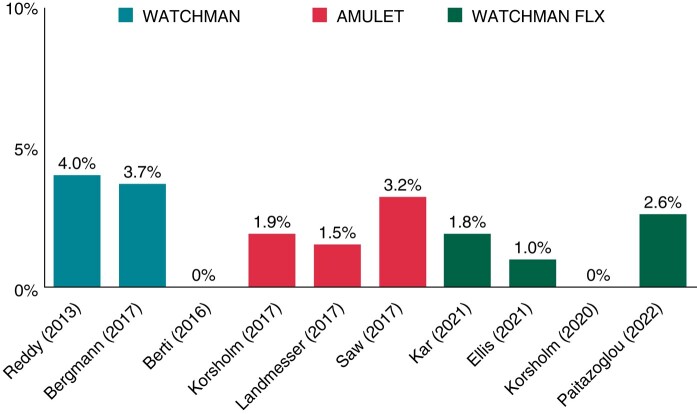
Incidence per 100 patient-years of DRT in LAAC registries with more than 100 patients.^86–95^

The incidence of DRT varies from 2 to 4%, although recent data with newer devices have reported a lower incidence of 1–2% per year (*Figure 7*).^86–95^ DRT is most frequently detected by routine imaging at scheduled follow-up visits after the procedure. It can be diagnosed with TOE or cardiac CT and it is associated with a 4–5 times higher risk of stroke/TIA.^96^ Besides patient-related risk factors, the risk of DRT can be increased by device implantation that is too deep resulting in incomplete LAA sealing.^97^ Hypercoagulability disorders, iatrogenic pericardial effusion, renal failure, and permanent AF are other risk factors for DRT.^96^ However, as new devices coated with thromboresistant fluorinated polymers are introduced, DRT should become rare and post-implant anti-thrombotic therapy may be simplified or eliminated.^98^

**Figure 8:**
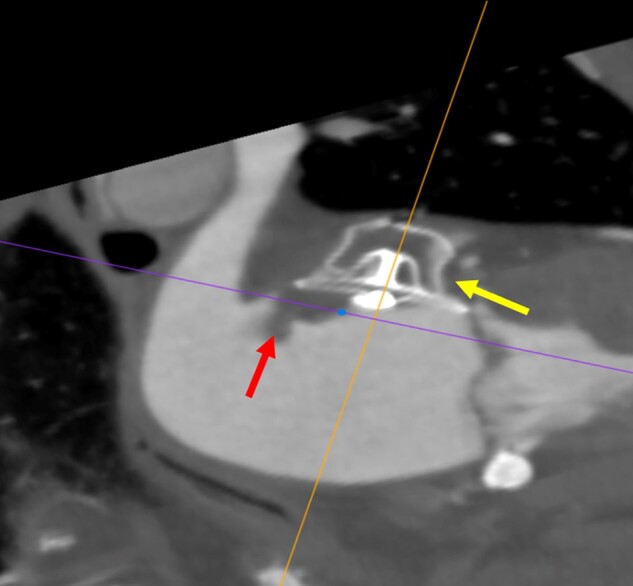
Device-related thrombosis (DRT) after LAA occlusion in a patient implanted with an AMULET device. The 3-month follow-up CT scan shows the AMULET device in a good position (leftward pointing arrow) with a large thrombus on the device disk (upward pointing arrow).

**Figure 9:**
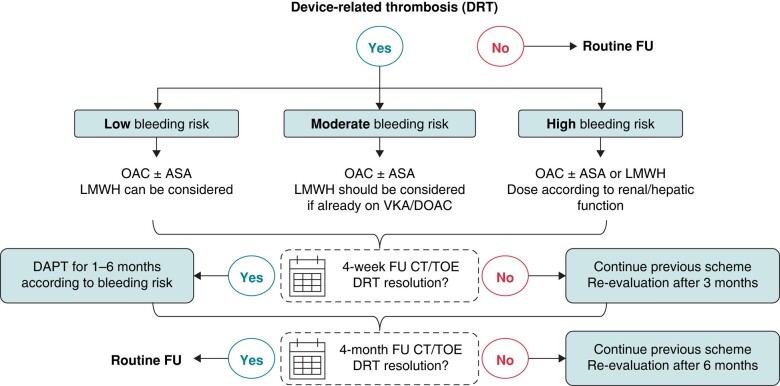
Flowchart showing an algorithm for treatment of DRT. DAPT, dual antiplatelet therapy; DOAC, direct oral anticoagulant; DRT, device-related thrombus; OAC, oral anticoagulant; FU, follow-up; LMWH, low molecular weight heparin; CT, computed tomography; TOE, transoesophageal echocardiogram; VKA, vitamin K antagonist.

Management of DRT usually implies escalation of anti-thrombotic therapy (low molecular weight heparin, LMWH, or DOACs), but this may be challenging or even harmful in patients who are at high bleeding risk. The common practice is to minimize time on anticoagulants until thrombus resolution is verified by imaging (*Figures 8* and *9*).

### Procedure-related stroke

During early experience, peri-procedural stroke occurred occasionally and mainly due to air embolism. However, nowadays peri-procedural stroke is a very rare event. In the SURPASS registry, the rate of all-cause stroke was 0.09% in hospital and 0.38% at 45 days.^81^ Procedural stroke/TIA may be related to the presence of thrombus/smoke in the LAA or LA, air embolization during the procedure, or development of thrombi on the delivery system or implanted device.

### Peri-device leak

**Figure 10:**
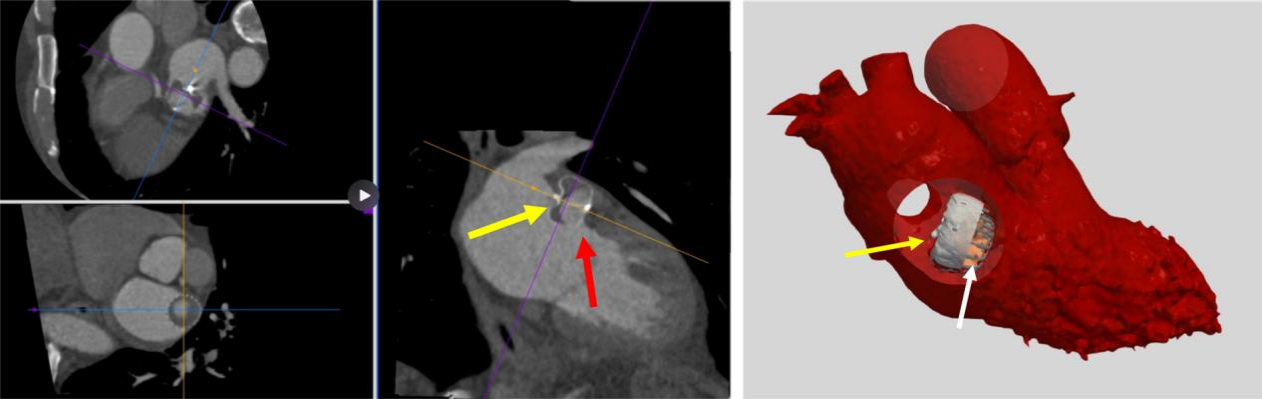
Follow-up CT scan (6 months) of a WATCHMAN FLX device that is not positioned correctly (middle panel rightward pointing arrow) showing a severe leak (middle panel upward pointing arrow). A 3D-segmented model (right panel) demonstrates that the device is rotated by 90°causing the leak at the inferior site of the device. CT, computed tomogram; TOE, transoesophageal echocardiogram.

**Figure 11:**
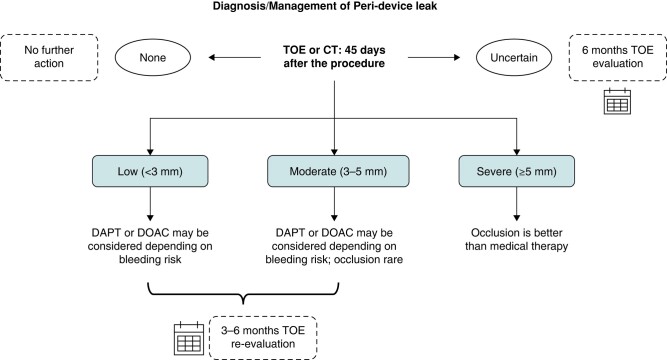
Flowchart showing a therapeutic approach when a peri-device leak is detected during follow-up. DAPT, dual antiplatelet therapy; DOAC, direct oral anticoagulants; TOE, transoesophageal echocardiogram.

The anatomy of the LAA is highly variable and can be very complex, including the landing zone for the LAA device, which is most often non-circular. Consequently, there is a risk of peri-device leak (PDL) after implantation or in some cases, a smaller lobe of the appendage may not have been occluded by the device.^99^ Peri-device leak can be diagnosed by TOE or even better with CT. With current procedural techniques and devices, small PDLs are rather frequent, whereas moderate leaks (3–5 mm) are less common and severe leaks (>5 mm) are very rare. Medical therapy is usually needed and is chosen according to bleeding risk. For PDL >5 mm, interventional leak closure with plugs, occluders, coils, or radiofrequency ablation may be considered but medical therapy may also be sufficient (*Figures 10* and *11*; Box 4).^100^

**Box 3:** LAAC: benefits, procedure and peri-procedural risk

Stroke prevention similar to OAC
No need for long-term OAC; reduced risk of bleeding
Procedure carried out in local analgesia/light sedation guided by ICE or micro-/mini-TOE
Procedure carried out in sedation/general anaesthesia guided by TOE
Duration of procedure: 30–60 min
Procedural risks:
Pericardial tamponade/effusion: 0.32–2.4%
Device embolization: 0.01–0.7%
Stroke: 0.09%
Death: 0.07%

## Special populations

**Figure 12:**
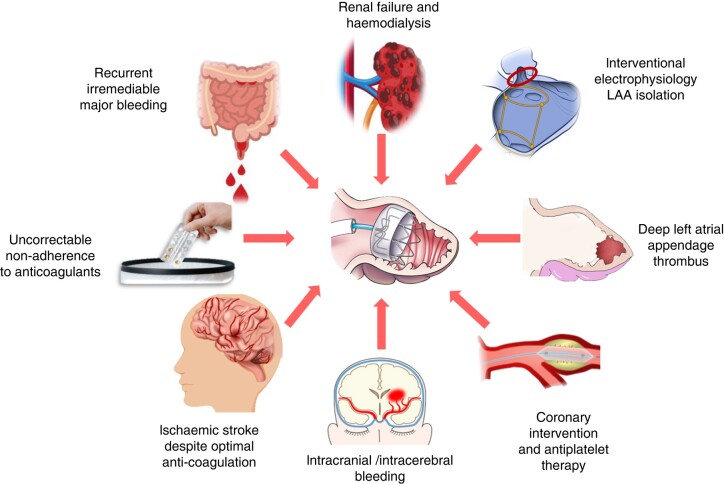
Clinical populations where LAAC may be considered for patients with AF at risk of stroke but refractory to or contraindicated for anticoagulation and when no otherwise satisfactory management is available.

There is a large range of medical circumstances in which LAAC therapy may offer an advantage over OAC (*Figure 12*). Many of these conditions may be associated with severe bleeding complications, ineffectiveness of anticoagulants against thromboembolism or patient adherence difficulties. Even minor bleeding may have severe effects, as for example, patients suffering from cerebral amyloid angiopathy.

Some ‘high-risk’ CV diseases may require the long-term use of antiplatelet therapy in addition to using an anticoagulant, to prevent new CV events such as re-infarction or stent thrombosis, but this comes at the expense of bleeding complications. If the use of OAC could be substituted by LAAC, the bleeding risk is mitigated, whilst stroke prevention is retained. Nonetheless, robust long-term data on this population group are lacking.

There are also patients that suffer a stroke or systemic thromboembolic event, or exhibit thrombus formation in the LAA despite using optimal anticoagulation therapy with an adequate INR or good drug compliance.

### Life-threatening or major gastrointestinal bleeding

Patients with AF and a high risk of stroke and embolism (CHA_2_DS_2_-VASc ≥2) who have a major bleeding event represent a highly challenging scenario, since effective chronic anticoagulation can be associated with a high or very high risk of recurrent bleeding. Hence, transcatheter LAAC was initially developed as an alternative mode for stroke prevention.^101^ One recent study suggested that only about 50% of patients with AF, admitted after a major or life-threatening bleeding are discharged with a plan for stroke prevention strategy, with only 10% being considered for LAAC.^102^

Nonetheless, a systematic review and meta-analysis found that restarting OAC therapy after a major bleeding event in AF was mostly associated with a positive clinical benefit when compared to not restarting OAC, with a significant reduction in any thromboembolism and all-cause mortality when resuming therapy no more than 2 weeks after gastrointestinal bleeding (GIB).^103^ For example, one study found that restarting OAC at discharge after GIB was associated with fewer thromboembolic events without a significantly increased risk of recurrent GIB at 90 days.^104^ Similar observations for reduced mortality and thromboembolism were seen in the Danish registries, although bleeding was higher in the long term.^105^ Nonetheless, the latter study was in the warfarin era, and contemporary studies with some DOACs suggest better GIB safety compared to warfarin.^106^ Hence, for many patients, the benefits of continuing anticoagulation (especially with DOAC) may outweigh the risks of recurrent GIB. Also, proton pump inhibitors may be protective in such patients.^107^ However, when GIB is associated with angiodysplasia, continuation of anticoagulation therapy may be such a high risk as to warrant consideration of other therapies such as LAAC.^108^

**Figure 13:**
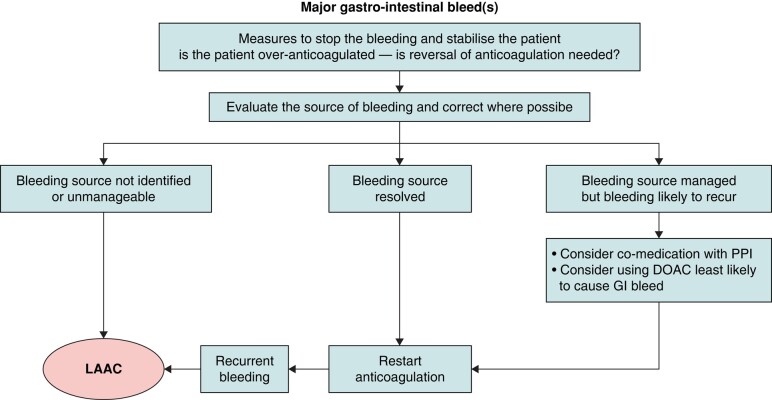
Management of (recurrent) major gastrointestinal bleeds. DOAC, direct oral anticoagulant; GI, gastrointestinal; INR, international normalized ratio; LAAC, left atrial appendage closure; PPI, proton pump inhibitor; TTR, time in therapeutic range; VKA, vitamin K antagonist.

Clinical registry studies have reported promising results in patients with AF and a high bleeding risk after LAAC.^16,109^ In the case of GIB, largely single-centre reports of LAAC have suggested its use as an alternative to OAC in patients presenting with major, recurrent or potentially unresolvable GIB (*Figure 13*).^108,110^ The multi-centre ACP registry reported their subgroup of patients with AF and previous major GIB, where LAAC was associated with a low annual rate of stroke/transient ischaemic attack, although peri-procedural major bleeding events were more frequent.^111^ Again, many of these studies were in the warfarin era, rather than with DOACs.

An important consideration in patients undergoing LAAC following a major or life-threatening bleed (especially from GIB) is the anti-thrombotic treatment regimen after LAA device implantation.^112^ This requires individualized decision-making, taking into account the patient's subsequent bleeding risk and the risk of device-associated thrombi, a recognized complication after LAA. In some clinical situations, particularly in patients with diffuse angiodysplasia, even a single antiplatelet drug may be enough to trigger recurrences of major haemorrhage. Given the greater biocompatibility of recent LAAC devices, earlier de-escalation of anti-thrombotic therapy is frequently performed in patients after major or life-threatening bleeding to avoid recurrent bleeding events.

### Cirrhosis and hepatic failure

Anticoagulants were contraindicated in patients with cirrhosis owing to concerns about bleeding risks, but recent studies have shown that patients with cirrhosis are not naturally anticoagulated and are at increased risk of prothrombotic events. Anticoagulant therapy may reduce the progression of hepatic fibrosis and be independently associated with increased survival and decreased decompensation.^113^

A higher incidence of AF has been observed in patients with cirrhosis, regardless of the underlying cause.^114^ There has been a 10% increase in the prescription of anticoagulants, primarily DOACs, for AF in patients with cirrhosis. The use of DOACs was associated with a lower risk of bleeding compared to warfarin.^115^ However, most available data are based on retrospective analyses and most studies included only a minimal number of patients with decompensated cirrhosis.

In cirrhotics with portal vein thrombosis, anticoagulation is associated with 9% bleeding complications in men,^116^ mostly not severe. However, the presence of severe thrombocytopenia <50.000 U/L (which is present in about 7% of patients) has been associated with increased bleeding complications with warfarin. Decompensated liver disease could be associated with more bleeding complications with OAC outside the indication for the treatment of PVT.^117^

Patients with severe portal hypertension can be more at risk of GI bleeding complications independently from variceal bleeding and often in this clinical setting, decompression of the portal system by intrahepatic portosystemic shunting is contraindicated by impaired cardiac function.

In cirrhosis, LAAC implantation has been associated with an increased cardiac tamponade and readmission rate compared to non-cirrhotic patients and GI bleeding seems to be responsible for this difference.^118,119^ Readmissions after the LAAC procedure are partially due to the prescription of antiplatelet therapy associated with concomitant chronic renal failure in about one-third of patients. Liver cirrhosis is a complex pathology, increasing both bleeding and thromboembolic risk. Careful patient selection and shared decision-making are critical for LAAO in cirrhotics due to increased complications and mortality. Pre-procedural optimization of haemostasis is necessary due to the increased bleeding risk.

### Intracranial haemorrhage

Stopping OAC and antagonizing the anticoagulant effect in patients with acute ICH is needed to reduce ICH-associated morbidity and mortality regardless of the presence of AF and the associated thromboembolic risk. In addition, surgical or catheter-based intervention may be needed in selected ICH patients. The residual risk of ischaemic stroke in non-anticoagulated AF patients is up to 15% per year, and about 80% of all ICH patients with AF are at high risk of ischaemic stroke. This underscores the need to manage thromboembolic stroke prevention after ICH.

Current evidence for the restarting of OAC after intracranial bleeding (ICB) is mainly based on prospective cohort studies and three RCTs, APACHE-AF,^120^ SoSTART,^121^ NASPAF-ICH,^122^ including no more than 340 patients in total.^123^ Taking these three RCTs together, restarting OAC was associated with reduced risk of ischaemic stroke on the one hand but increased risk (of borderline significance) for recurrent ICH.^124^ The threat of ICH recurrence is daunting but many physicians will consider restarting anti-thrombotic therapy at least 30 days after the ICH.^125^ The results of ongoing RCTs focusing on OAC vs. no anticoagulation (without considering LAAC) in ICB patients with AF (such as ENRICH-AF,^126^ PRESTIGE-AF,^127^ A_3_ICH,^128^ STATICH,^129^ and ASPIRE) are awaited.^130^

Despite the fact that there is no proven benefit of LAAC in ICH patients according to a RCT so far, LAAC is recommended by AF guidelines^52,131^ and consensus papers worldwide.^132^ Publications based on propensity score-matched analyses in AF patients with ICH undergoing LAAC vs. medical treatment conclude a benefit of LAAC regarding the composite of ischaemic stroke, major bleeding and all-cause mortality.^40,41^ At present, moderate-sized RCTs comparing LAAC to OAC/best medical treatment exclusively including ICH patients such as CLEARANCE,^30^ and STROKE-CLOSE,^29^ or patients at very high risk of bleeding including ICH patients, such as CLOSURE-AF^28^ are ongoing. Special attention has to be paid to ICH patients with (suspected) cerebral amyloid angiopathy, refractory hypertension or concomitant chronic renal failure (including those on dialysis), who might benefit most from LAAC and such studies are underway (SAFE LAAC CKD,^133^ LAA-Kidney.^33^)

In clinical practice, LAAC after ICH has ‘an acceptable peri-procedural and post-procedure risk’ according to expert consensus.^134^ Of note, restarting of antiplatelet therapy (as needed after LAAC) is safe after ICH as demonstrated in the RESTART study, randomizing patients on anti-thrombotic therapy for the prevention of occlusive vascular disease at the time of ICB to restarting or avoiding antiplatelet therapy.^134^ However, it remains to be established in RCTs such as CLOSURE-AF whether stopping antiplatelet(s) several months after LAAC is safe or associated with increased risk of thrombus formation and (subsequent) stroke in AF patients and prior ICH.

### Ischaemic stroke in atrial fibrillation patients whilst on an oral anticoagulant

There is a surprising shortage of evidence of evidence regarding efficacy and safety of LAAC compared to OAC in secondary stroke prevention. The RCTs focusing on LAAC vs. medical therapy (such as PROTECT-AF, PREVAIL, and PRAGUE-17) and even large prospective LAAC registries (such as LAARGE, EWOLUTION, AMULET observational registry) did not focus on AF patients after ischaemic stroke. However, residual stroke risk in anticoagulated AF patients is about 1–2% per year in RCTs and may be even higher in clinical practice and in secondary stroke prevention. In the prospective Berlin AF Registry, about 60% of all registry patients with known AF were on OAC at the time of the index-stroke or TIA.^135,136^ Of note, under-dosing of DOAC/VKA or a competing stroke aetiology (besides AF) is a frequent finding in AF patients with acute ischaemic stroke or TIA.^136,137^ However, a pooled observational cohort study underlines that about half of all AF patients with ischaemic stroke whilst taking an OAC are neither under-dosed nor have a competing stroke mechanism.^137^

As demonstrated by the COMBINE-AF investigators,^138^ and by multi-centre observational RENO-EXTEND study,^139^ there is a relevant recurrent stroke risk and a rather high mortality rate after ischaemic stroke whilst on OAC. Interestingly, a pooled analysis of observational cohort studies did not demonstrate a benefit of changing the type of OAC^140^ or changing DOAC treatment in secondary stroke prevention or adding an antiplatelet on top of OAC.^137^

**Figure 14:**
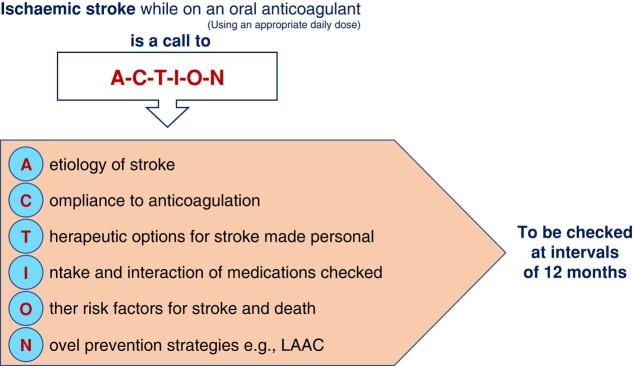
A-C-T-I-O-N items that should be considered in AF patients suffering an ischaemic stroke whilst on an anticoagulant.^141^

Therefore, AF patients suffering an ischaemic stroke whilst on DOAC therapy (properly dosed and taken adherently) are a call to A-C-T-I-O-N, (*Figure 14*) referring to A—*Aetiology of stroke revisited?*, C—*Compliance to OAC optimized?*, T—*Therapeutic options in secondary stroke prevention personalized?, I—Intake and interactions of present medication checked?*, O—*Other risk factors for stroke or death treated?* and N—*Novel stroke prevention strategies available?*^141^

Because of a significant residual risk of stroke under anticoagulation (that may be estimated to be 7% at 1 year and 10% at 2 years), novel stroke prevention strategies may include LAAC.^138^ In an international collaboration of LAAO registries (STR-OAC), a propensity score-matched comparison between those treated with LAAC compared to those managed by the standard of care, the LAAC cohort was associated with fewer subsequent ischaemic strokes.^142^ LAAC on top of OAC therapy may also be worth considering in light of the results of the randomized LAAOS-III trial demonstrating risk reduction of stroke and SE after surgical LAAC in AF patients undergoing heart surgery and continuing OAC afterwards.^22^ Prospective RCTs using catheter-based LAAC on top of OAC vs. OAC are underway and will hopefully start enrolment soon (LAAOS-4;^143^ ELAPSE^144^).

Further novel prevention strategies may include early rhythm-control therapy in addition to OAC,^145^ left atrial catheter ablation on top of DOAC treatment (as in the ongoing randomized STABLED trial^146^), bilateral permanent percutaneous carotid artery filter^147^ on top of DOAC treatment (as in the planned randomized INTERCEPT trial^148^) or, if and when approved, a Factor XIa inhibitor form of OAC.

### Left atrial appendage thrombus despite optimal oral anticoagulant

Despite optimal OAC treatment, thrombus formation may be detected in the LAA in patients with AF. The current recommendations suggest that LAAC should not be performed, because of the high risk of promoting dislodgement of the thrombus and, thus potential cerebral and SE. Therefore, the therapeutic options in this category of patients are limited. On the other hand, the presence of thrombus in the LAA *per se* is considered at high risk of favouring ischaemic stroke and TIA.^149–151^ In a recent meta-analysis, the prevalence of left atrial thrombus in patients with AF or atrial flutter during optimal anticoagulation was 2.7%, regardless of whether patients were treated with a VKA or DOAC.^152^

The management of these patients is usually challenging, ranging from reaching a higher INR in patients treated with a VKA, switching one DOAC drug to another, to adding antiplatelet medication to VKA or DOAC treatment. Alternatively, also using LMWH or unfractionated heparin in combination with aspirin or clopidogrel was reported.^52,151–153^ Notably, these approaches result in the dissolution of thrombus only in 42.6% of cases.^154^ This indicates the need to devise alternative modalities of treatment for patients with resistant LAA thrombus,^155^ particularly after LAAC electrical isolation.^156^

**Figure 15:**
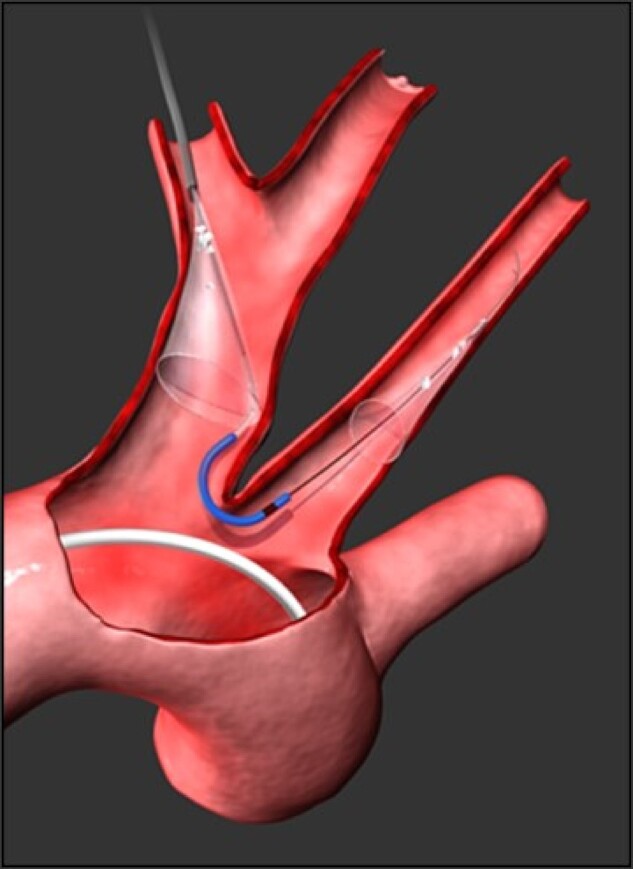
Diagram illustrating positioning of the Sentinel™ cerebral protection filter system (Boston Scientific, Marlborough, MA, USA). The system is designed to protect the cerebral vasculature from embolic events and remove debris/thrombus during interventional procedures, such as TAVI, but it has been used for LAAC in patients with thrombus formation in LAA. The device comprises dual-filter embolic protection and is percutaneously placed in the aortic arch. The two self-expandable filters directed into the carotid arteries can adapt to a wide variety of anatomies and have the ability to block even debris of less than 0.5 mm in size.

The use of LAAC in case of thrombus formation in the LAA is anecdotal^157,158^ and even if formally contraindicated by the current guidelines, there is neither any formal agreement nor technical indication. One of the main aspects is the differentiation between fresh and old thrombus, the latter being more manageable. The anatomic location is also important since an old thrombus deep in the LAA might be more organized and considered less prone to be dislodged and provoke an ischaemic event during LAAC. If LAAC is considered in a patient with LA thrombus, the first crucial step is to ensure cerebral protection during the procedure, e.g. using Sentinel (Boston Scientific, Marlborough, MA, USA), to minimize the risk of intra-procedural ischaemic events (*Figure 15*).

### Coagulation disorders

Disorders of haemostasis have a double-sided relation with LAAC: as an increased risk for bleeding, they may represent an indication for LAAC—at the same time they also represent a bleeding risk during implantation and during subsequent anti-thrombotic treatment. Haemorrhagic disturbances occur due to:

- impaired number or function of platelets,
- deficiencies of coagulation factors, and
- vasculopathy such as angiodysplasias or increased capillary fragility.

All of these may be either congenital or acquired. Some of those patients may develop a thromboembolic risk in spite of their coagulation disorder, particularly with increasing age, which then may necessitate stroke prevention if AF develops (see below).

If a relevant bleeding disorder is identified, a treatment plan for LAAC and the subsequent anti-thrombotic treatment should be provided by a coagulation expert working with a LAAC implant specialist. Most mild bleeding disorders respond to desmopressin and/or antifibrinolytic drugs, regardless of aetiology. Platelet function disorders also require specialist management.^159^

#### Important practical issues

Von Willebrand's disease (VWD) is the most common congenital haemorrhagic disorder. Acquired VWD can be due to consumption/destruction of von Willebrand factor (VWF) in patients with valvular stenosis or artificial valves, also in patients with myeloproliferative neoplasia. Von Willebrand's disease cannot be excluded by an activated partial thrombin clotting time and prothrombin time test. Thromboembolic complications may occur in VWD, particularly in mild VWD and/or because VWF generally increases with age. The indication for anticoagulation should be discussed within an MDT appreciating the overall risks, including bleeding history, relevant bleeding scores, laboratory findings and CHA_2_DS_2_-VASc score.

#### Indication for left atrial appendage closure implantation in haemostatic disorders

Von Willebrand factor typically increases with age in Type 1 VWD, so that these patients may require thromboembolic protection in case of AF. Anticoagulation could be considered if VWF has returned to the normal range and the bleeding history has been negative for at least the last decade. In other types of VWD, or low VWF or a positive bleeding history, LAAC can be considered. This may also apply to myeloproliferative disorders, which can lead to acquired VWD and/or impaired platelet function. The same considerations apply to patients with a reduction of single coagulation factors, in which the therapeutic decisions between anticoagulation and LAAC should also be made by an MDT with cardiology and haemostaseology expertise.

Patients with vasculopathies such as Rendu-Osler-Weber hereditary telangiectasia suffer from repetitive bleeding, most prominently from the nasopharyngeal tract, and although this may sometimes be acutely solvable by cauterization, it is often recurring and exacerbated using platelet-inhibitors and anticoagulants. More severe arterio-venous malformations may exist in the lungs, intestine, bladder, and brain, which may also lead to major bleeding events and may not be solved so easily without an arterial coil or endoscopic cauterization operation that carries substantial risk. Although the bleeding impact may not always be severe, its repetitive nature bringing discomfort to the patient is justification enough to not make it worse by using long-term anticoagulation, if indicated otherwise.

### Severely reduced glomerular filtration rate and kidney failure

The prevalence of AF is high in patients with an estimated glomerular filtration rate (eGFR) between 15–29 mL/min (stage chronic kidney disease, CKD, G4) and <15 mL/min not on dialysis (stage CKD G5) or undergoing dialysis (stage CKD G5D). The United States Renal Data System (USRDS) reports that about one out of four CKD G4–5 and G5D have AF.^160^ The finding is probably underestimated, particularly in the haemodialysis (HD) population, because of the high rate of intra-dialytic AF episodes that often remain undiagnosed.^161^ An HD session can also trigger arrhythmia because of the often large and abrupt intra-dialytic volume and electrolyte changes.^162^

**Figure 16:**
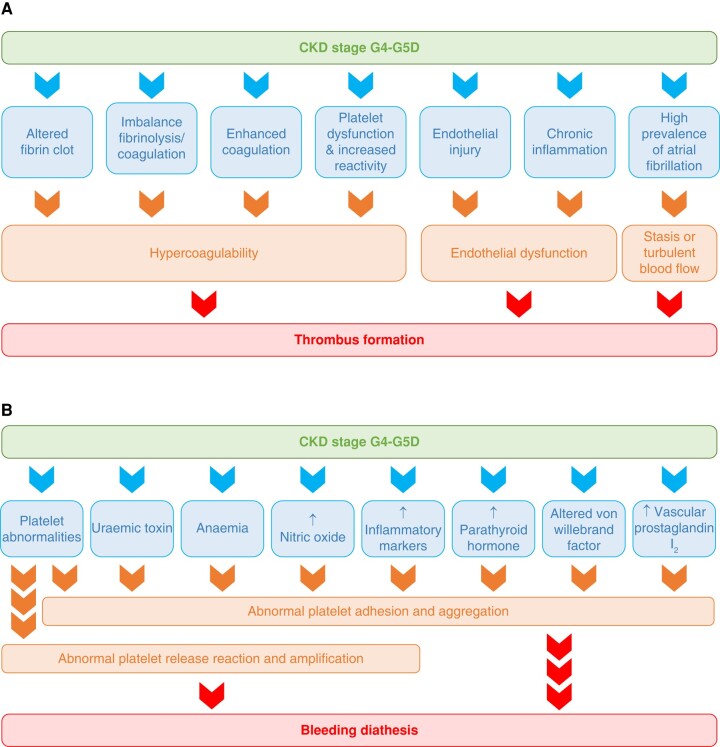
Diagrams illustrating the prothrombotic (*A*) and pro-haemorrhagic (*B*) tendencies seen in severe chronic kidney disease. CKD, chronic kidney disease; G4–G5D, grade of severity of CKD (modified from Ng et al.^163^).

Thromboembolic and haemorrhagic risks are elevated in patients with very low eGFR. Both prothrombotic factors (the presence of endothelial dysfunction and hypercoagulability; *Figure 16A*) and factors promoting bleeding (abnormal platelet adhesion and aggregation and abnormal platelet release reaction; *Figure 16B*) are simultaneously present.^163^

Atrial fibrillation is associated with a worse prognosis in terms of all-cause and cardiovascular death in patients with reduced eGFR and kidney failure, as in the general population.^164,165^ United States Renal Data System reports adjusted 2-year survival probabilities of 55.1% in HD patients with AF and of 72.1% in those without AF.^160^

There are several uncertainties and difficulties in treating these patients. Randomized controlled trials demonstrating the efficacy of VKA for thromboembolic prevention are lacking and observational studies in HD patients have yielded uncertain results on VKA efficacy and negative results on safety.^166^ As eGFR worsens, the INR time in the therapeutic range (TTR) decreases, leading to an increased risk of bleeding.^167,168^ Vitamin K antagonists are also known to increase the risk of vascular calcifications,^169^ which is an important issue in uraemic patients, already particularly prone to this CV complication. The presence of eGFR < 25–30 mL/min was an exclusion criterion for recruitment in DOAC vs. VKA Phase III RCTs.^170–173^ Two recent meta-analyses of studies performed in severely reduced eGFR and kidney failure populations were unable to demonstrate that OAC therapy (both VKAs and DOACs) was associated with a reduced risk of thromboembolism.^174,175^

Neither cardiology nor nephrology guidelines have been able to provide clear guidance on what is the best treatment for a patient with AF and eGFR < 15 mL/min.^132,176^ Therefore, nephrologists often decide not to prescribe OAC therapy to their patients or discontinue the drug after major bleeding.^177^

Left atrial appendage closure may be a valuable alternative for treating these patients. Limited data, derived largely from retrospective registry studies, are available in CKD G4–5 and G5D patients undergoing the procedure. Overall, these studies show an increased in-hospital and long-term mortality risk in patients with severely reduced eGFR and kidney failure compared with those with preserved renal function who underwent the procedure. However, no significant differences were reported between the two populations in terms of thromboembolic and bleeding events incidence.^178–183^ WATCH-HD which employed both retrospective and prospective registry data demonstrated that LAAC was a safe and effective therapy for carefully selected HD patients.^184^

Data comparing the efficacy and safety of LACC vs. OAC therapy are very few in patients with stage CKD G4–G5D. Two RCTs evaluating the safety of LAAC vs. OAC therapy in patients with eGFR <30 mL/min WATCH AFIB,^185^ and STOP-HARM,^186^ were terminated prematurely due to failure to recruit patients.^187^ However, another RCT, LAA-KIDNEY,^33^ recently started and recruitment is ongoing. The only prospective study that included a fair-sized sample of dialysis patients showed a reduction in thromboembolic events in patients undergoing LAAC with respect to the events observed in both a cohort of dialysis patients with AF not taking OAC therapy and a cohort of patients taking warfarin. The risk of bleeding in the LAAC cohort was lower compared to the Warfarin cohort, whilst there were no significant differences between the LAAC and the cohort not taking any therapy. Nearly half of the bleedings occurred in the first 3 months after the procedure, when most patients were taking dual antiplatelet therapy.^188^ Post-LAAC anti-thrombotic therapy is also currently being investigated in the SAFE LAAC CKD trial.^133^

**Figure 17:**
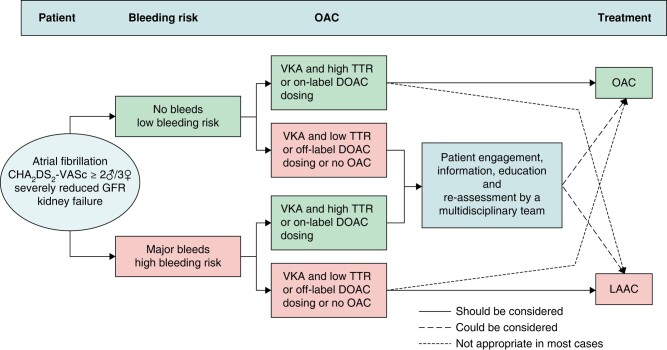
Proposed algorithm for treatment choice in patients with severely reduced glomerular filtration rate and kidney failure. OAC, oral anticoagulant therapy; DOAC, direct oral anticoagulant; GFR, glomerular filtration rate; LAAC, left atrial appendage closure; TTR, time in therapeutic range; VKA, vitamin K antagonist.

Whilst awaiting the results of further studies in CKD G4 and G5D patients with a high risk of AF-related stroke, it is reasonable to evaluate the use of anti-thrombotic therapies in the context of the individual's stroke and bleeding risk. Certainly, for those patients who have a high bleeding risk, especially if they have already sustained a major or life-threatening bleed, or are incapable of taking OAC, LAAC therapy is a possible therapy (*Figure 17*). Similarly, for those who have a low bleeding risk and can take OAC without difficulty, OAC is the therapy of choice and LAAC is inappropriate. In other situations, the choice between LAAC and OAC is less clear and highly patient-dependent.

### Prolonged dual antiplatelet therapy

A previous history of CV disease and myocardial infarction is prevalent in about 10% of patients with AF.^189,190^ Incident myocardial infarction increases the risk of mortality.^191^ In order to prevent arterial thrombotic events, patients with complex coronary artery disease, e.g. acute coronary syndrome (ACS) and percutaneous coronary intervention (PCI) require antiplatelet therapy. In the acute phase, intensified inhibition of platelet function, commonly as dual antiplatelet therapy including aspirin and a P2Y12 inhibitor is most effective. In combination with OAC in AF patients, bleeding risk remains very high even with DOAC therapy.^192–195^ With a single antiplatelet therapy in combination with DOAC, the risk of stent thrombosis is mildly elevated.^196,197^ Therefore, patients with high ischaemic risk, e.g. recurrent coronary events, multi-vessel or complex stenting, prior stent thrombosis may require prolonged dual antiplatelet therapy.

The relevance of dual antiplatelet therapy has been shown in a sub-analysis of the AUGUSTUS trial: maintaining aspirin in the anti-thrombotic regimen as triple therapy for 1 month after PCI or ACS is beneficial to reduce ischaemic events at a high risk of bleeding (7.45%).^198^ In addition, timely de-escalation in the ambulatory setting is often not performed.^199^ Previous ESC/EACTS guidelines stated that percutaneous LAAC may be considered in patients at high stroke risk and contraindication for long-term combined antiplatelet and OAC therapy (Class IIb, level of evidence B).^200^

The choice of LAAC rather than OAC in high bleeding risk patients needing prolonged therapy with antiplatelet therapy may offer the opportunity to reduce or stop OAC. First, small studies have examined LAAC in combination with PCI.^201,202^ Performing the procedures in 24 ACS patients with AF in the same session may be feasible.^201^ In a Korean cohort study that compared 41 AF patients undergoing drug-eluting stent implantation with LAAC and dual antiplatelet therapy with 434 patients on dual pathway inhibition could show better net clinical outcomes for cerebrovascular and major bleeding events in the occluder group. Two ongoing studies are investigating the role of LAAC in patients with complex coronary artery disease and PCI in comparison with DOAC-based anti-thrombotic regimens.^203,204^

### Left atrial appendage closure during/after other cardiac interventions

Since LAAC is a preventive intervention, it may be considered when another procedure is performed in the left atrium, thereby offsetting procedural complications of a separate intervention. In addition, workflow and cost-effectiveness optimization may be improved in this context. The argument for combining interventions is analogous to the rationale studied in the LAAOS-III trial where patients undergoing cardiac surgery (and thus exposed to the risks of surgery anyway) experienced a clear stroke risk reduction without an increase in undesirable outcomes if surgical LAAC was performed during the procedure.^22^ On the other hand, both procedures must be independently indicated, and LAAC is not indicated simply because another procedure is taking place.

The very favourable evolution of contemporary LAAC complication risks, as outlined elsewhere in this document, makes this argument viable in the setting of several other routine cardiac interventions. Specific considerations may exist for specific procedure types as outlined below.

#### Left atrial ablation

A high rate of OAC discontinuation after AF ablation is seen in several studies, despite an increased stroke risk associated with discontinuation after 3 months in patients with CHA_2_DS_2_-VASc ≥ 2.^205^ Current guidelines, therefore, recommend continuing OAC indefinitely in these high-risk groups. A strategy combining AF ablation and LAAC for the purpose of allowing OAC cessation appears attractive and has been shown to be safe and efficient without interference when a repeat ablation is needed.^206,207^ A small proof-of-concept RCT comparing LAAC to warfarin post-ablation showed no events in either group.^208^ Whether there is a net clinical benefit of such a strategy as compared to contemporary DOAC continuation as per current guidelines is the subject of the OPTION RCT.^35^

Conversely, arguments can be made for a staged approach to ablation and LAAC (typically in that order although not necessarily so). First and foremost, an apparently successful AF ablation may reduce stroke risk although existing evidence for this is sparse. Formal testing of OAC vs. aspirin alone is being conducted in the OCEAN trial.^209^ In addition, concerns exist regarding the location of the transseptal puncture site, which may be suboptimal for LAAC in the typical pulmonary vein isolation (PVI) positions. The presence of ablation-induced oedema at the LAA-left pulmonary vein ridge immediately after ablation may occasionally lead to sizing errors and to suboptimal occlusion during follow-up.^210^

#### Left atrial appendage electrical isolation

There is conflicting evidence for electrical isolation of the LAA to improve catheter ablation outcomes. The aMAZE randomized trial failed to show a rhythm-control benefit of LAA exclusion and isolation over PVI alone.^211^ However, the BELIEF-RCT and several observational studies showed improved rhythm control.^212^ For the latter, strategies of LAA isolation without LAA exclusion (i.e. not using surgery or the LARIAT device), there is an additional concern regarding increased stroke risk after LAA isolation (intentional or not) even for patients on OAC, due to loss of LAA mechanical function.^213^ Firm recommendations on the usefulness of LAA isolation are not available at this point, although there does appear to be growing consensus to recommend LAAC in case of electrical isolation.^214^

#### Transcatheter aortic valve replacement and left atrial appendage closure

Transcatheter aortic valve implantation (TAVI) has emerged as the standard treatment modality for patients with severe aortic stenosis across the full risk spectrum. Atrial fibrillation occurs in more than 10% of octogenarians and is the most common arrhythmia in the TAVI population, being present in about 30–40%. Typically, TAVI patients are older than 75 years with multiple comorbidities. In patients with AF undergoing TAVI, bleeding complications were reported to be as high as 50%, and in those who experience bleeding complications during the first year, 1-year mortality is doubled.^215,216^ Left atrial appendage closure-obviating the need for OAC may therefore be an attractive treatment for the AF TAVI population.

Current evidence remains limited to only a handful of observational and prospective studies.^217,218^ Limited data indicate that a combined TAVI-LAA closure intervention is a feasible and potentially effective approach for stroke prevention in patients with symptomatic, severe AS and AF with a high bleeding risk. Larger randomized trials with longer follow-up are needed to confirm safety and to further show the efficacy of combining these two increasingly common interventions.

#### Transcatheter mitral valve edge-to-edge repair and left atrial appendage closure

Patients undergoing transcatheter mitral valve edge-to-edge repair (TEER) are frequently affected by AF and are at high risk for major bleeding due to comorbidities or concomitant indications for anti-thrombotic therapy. From a procedural aspect, there are similarities. Transcatheter mitral valve edge-to-edge repair and left atrial appendage closure are performed via the femoral venous route and both require a similar transseptal crossing, hence it seems reasonable to combine them. Currently, available evidence on simultaneous or successive TEER and LAAC is very limited, derived from case reports and very small case series,^219–224^ with short follow-up, showing high immediate technical success and an acceptable rate of major complications as well as in the long-term comparable efficacy (stroke, death) and safety (major bleeding). With TEER becoming more and more mainstream therapy, there is a need for larger prospective studies to address the potential of these therapies to be performed simultaneously or successively.

#### Left atrial appendage closure and other concomitant cardiac interventions (percutaneous coronary intervention, atrial septal defect, patent foramen ovale closures)

There is very limited reporting of LAAC performed as a simultaneous procedure with PCI and also with atrial septal defect closures.^201,225^ Similar procedural outcomes were reported for isolated LAA closure procedures and the combined procedure.^226^ At the current state of knowledge, such interventions should only be carried out on an individual basis with prior careful assessment by the structural heart team. To be applied more widely, validation in larger studies is needed.

### Patient refusal/non-adherence/non-compliance

Physicians may decide not to prescribe OAC to patients who fall or are frail or instead they may offer treatment with OAC at doses less than those that are effective.^227^ Patients may refuse OAC because of relatively mild bleeding or because they hear from their friends and neighbours that the therapy is dangerous. Others may be completely averse to taking regular medication especially when it is preventive rather than directed at symptoms which are troubling the patients. Even when patients receive and accept appropriate prescriptions, evidence suggests that a high proportion of patients no longer persist with their medication or frequently lapse from their therapy, leaving them at risk for stroke.^228^ A recent meta-analysis on adherence showed that adherence/persistence to DOAC was particularly poor: one-third of AF patients starting DOAC stopped the drug by 1 year, and another third of patients were taking the DOAC less than 80% of the time.^229^ Elderly patients, especially those with physical disabilities or mental illness, may need to rely on others to ensure optimal adherence and such a supportive social framework is often not readily available. In these patients, LAAC may provide an alternative treatment that is not limited by such compliance issues.

**Figure 18:**
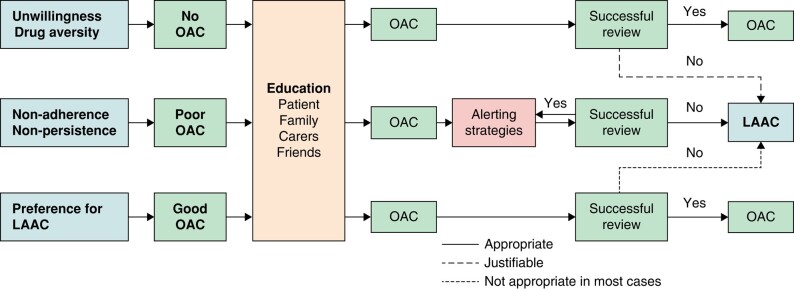
Management of refusal/non-compliance/non-persistence with OAC therapy and use of LAAC. The patient may be averse to oral anticoagulant therapy, non-compliant or simply prefer LAAC therapy. In these cases, the physician and other health care professionals are expected to educate the patient, the family and/or carers and friends. The patient may resume or improve compliance in which case anticoagulant therapy should continue, but if best efforts fail a LAAC device may be the best solution. OAC, oral anticoagulant; LAAC, left atrial appendage closure device.

For patients treated with VKA, regular assessment of the INR easily reveals those whose therapy is inadequate but for those taking DOACs prescription monitoring, pill counting, and the recollections of patients or their carers is usually all there is to assess how well the oral anticoagulation regimen is being followed. A counselling programme might be started to help the patient understand the value of the treatment and how important it is to follow the prescription. When patients cannot be relied on to take their medications regularly, a LAAC device may be preferable (*Figure 18*).

Also, if the patient is rigidly drug therapy averse, LAAC therapy can be considered, provided that the patient is willing to use anti-thrombotic medication for a limited period after implantation of the device. It is also relevant to be sure that the patient has no other life-threatening comorbidities that require continuous drug therapy which might be refused.

Patients may learn about LAAC therapy and simply prefer this option to taking regular anticoagulant drugs. This is often the case when the patient has been referred for consideration of LAAC implantation and has been informed about some of the advantages of this therapy. It may then be very difficult to re-align the patient toward anticoagulant therapy. However, this should be attempted because there is still only limited evidence that LAAC is as beneficial as DOAC therapy. The 2023 ACC/AHA/ACCP/HRS guidelines do accept that patient preferences may be considered when there is a high risk of stroke and of bleeding (a Level IIb recommendation—see above).^56^

## Anticoagulant/antiplatelet therapy regimens after left atrial appendage closure

**Figure 19:**
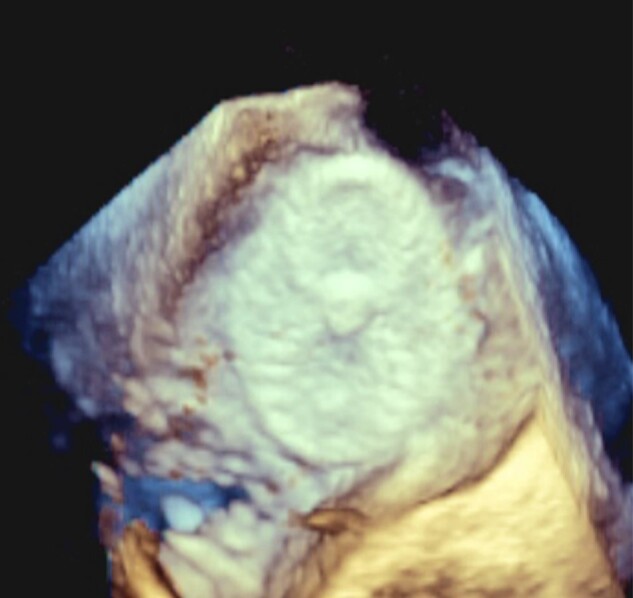
3D echocardiogram, demonstrating endothelium growing over the device which was implanted 7 weeks previously.

Anti-thrombotic therapy is required after LAAC in order to prevent device-related thrombus and this is of special importance in the initial phase, before device endothelization (*Figure 19*).^62,230,231^

Published data on anti-thrombotic regimens were derived from studies performed on patients who were eligible for anticoagulation (who received VKA or DOAC), as well as from studies performed on patients with intolerance or relative contraindications to anticoagulation, mainly related to prior major bleeding complications (who received antiplatelet therapy).^230^

Clinical RCT data on patients without LAAC have shown that dual antiplatelet therapy with aspirin–clopidogrel had similar major bleeding and ICH rates to warfarin (ACTIVE-W).^232^ When aspirin was compared to apixaban in AF patients who refused or were deemed ineligible for warfarin, there was clear superiority of apixaban for the reduction of stroke/SE but the rates of major bleeding and ICH were similar (AVERROES).^233^ In the BAFTA trial of elderly (age ≥75 years) AF patients managed in primary care, aspirin monotherapy had similar rates of major bleeding or ICH as warfarin.^234^ In elderly AF patients with high-risk features for bleeding, low-dose edoxaban 15 mg was superior for stroke risk reduction, with a non-significant difference in major bleeding or ICH to placebo, although major GI bleeding was increased with edoxaban (ELDERCARE-AF).^235^

**Figure 20:**
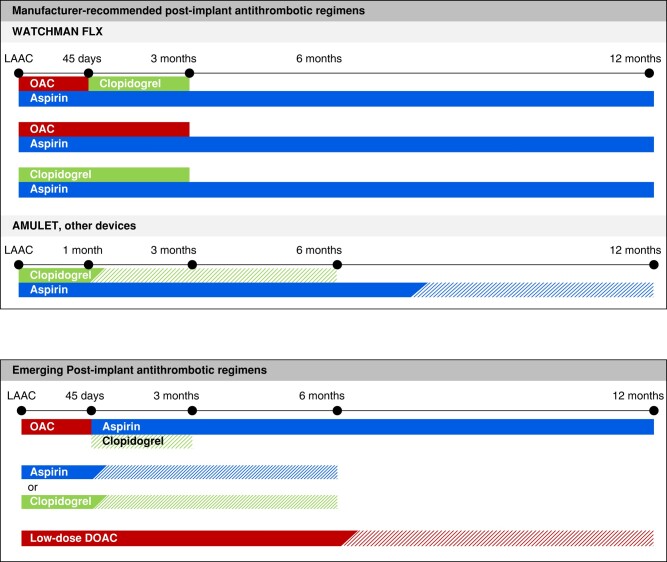
Upper panel: Manufacturer-recommended anti-thrombotic regimens after LAAC (adapted and updated^237,238^). LAAC, left atrial appendage closure; OAC, oral anticoagulant. Lower panel: Emerging strategies for anti-thrombotic regimens after LAAC (limited evidence and some ongoing studies): initial anticoagulant without concomitant aspirin^239–241^ followed by a DAPT or SAPT period; single antiplatelet^242–245^; low-dose DOAC^246–250^. LAAC, left atrial appendage closure; (D)OAC, (direct) oral anticoagulant. Hatching indicates variable adoption depending on benefit–risk.

In practice, after LAAC there is a need to tailor the anti-thrombosis regimen according to the patient. The best anti-thrombotic therapy after LAAC needs to provide a balance between the prevention of DRT and the occurrence of major bleeding. The rationale for choosing between the available options (*Table 9* and *Figure 20*) should be based on physician assessment of individual patient characteristics, such as bleeding risk and stroke risk, an overall clinical evaluation of the patient's condition, comorbidities and preference, as well as an evaluation of the reasons for LAAC.^61,62,236^ As reported in *Table 9*, discontinuations of OAC or antiplatelet therapy after LAAC is subject to the absence of other clinical indications for that medication and an assessment, including proper imaging (TOE or CT), demonstrating that there are no significant peri-device leaks (>5 mm), thrombus on the device or recent history of clinical events. Currently accepted anti-thrombotic regimens are illustrated in *Figure 20*.

**Table 9:** List of main anti-thrombotic schemes used after LAAC

Anti-thrombotic regimen	Supporting studies	Main scheme
VKA^a^	PROTECT-AF, PREVAIL, AMULET IDE	Aspirin + VKA (INR 2.0–3.0) for at least 45 days post-implant Aspirin + clopidogrel from 45 days until 3 months post-implant Then aspirin alone until 12 months post-implant
DOAC^a^	PINNACLE-FLX, EWOLUTION	Aspirin + DOAC for at least 45 days post-implant Aspirin + clopidogrel from 45 days until 3 months post-implant Then aspirin alone until 12 months post-implant
Dual antiplatelet	ASAP, EWOLUTION, AMULET Registry, AMULET IDE	Aspirin + clopidogrel until 3 months (WATCHMAN FLX) or 6 months (AMULET) post-implant Then aspirin alone until 12 months post-implant

DOAC, direct oral anticoagulation; INR, international normalized ratio; LAAC, left atrial appendage closure; VKA, vitamin K antagonist.

^a^Oral anticoagulant schemes are not recommended with the AMULET device unless residual flow around the device is >5 mm.

In a pooled analysis of data on patients from the PROTECT-AF, PREVAIL, CAP, CAP2, ASAP and EWOLUTION studies, patients receiving either oral anticoagulants or antiplatelets post-LAAC implant were matched and compared with regard to the occurrence of non-procedural bleeding and stroke/systemic thromboembolism over 6 months following implantation. Although DRT was more frequently observed with antiplatelet therapy, the occurrence of major bleeding and of stroke/systemic thromboembolism was similar between regimens based on antiplatelets or OAC.^251^  *Figure 20* shows various manufacturer recommendations and less ‘official’ strategies for anti-thrombotic therapy post-implant.^237–250^

Observational data from the years 2016–2018 in the United States highlighted how the anti-thrombotic regimen approved by the FDA for use of the WATCHMAN device was rarely applied.^239^ In particular, discharge after implantation on VKA or DOAC without concomitant aspirin was common and associated with lower risk of adverse outcomes. Updated data were presented at the HRS conference in 2023, confirming this finding.^240^ In a recent meta-analysis comparing initial anti-thrombotic therapy following LAAO, monotherapy with DOAC had the highest likelihood of lower thromboembolic events and major bleeding.^241^

A simplified regimen with a short period (2–4 weeks) of a single antiplatelet (acetyl salicylic acid or clopidogrel) has also been applied to very selected patients with an extremely high bleeding risk on the basis of expert consensus,^62^ and reported in observational studies.^242–244^ Additional data on this approach may become available from the CLOSURE-AF^28^ and the ARMYDA-AMULET^245^ ongoing studies.

Limited but promising observational data are available on post-LAAC treatment with low-dose DOACs, showing reduction of DRT, thromboembolism, and major bleeding events compared with a standard, antiplatelet-based, anti-thrombotic therapy;^246,247^ however, further controlled data are required to assess the value of this strategy. The small randomized ADALA trial^248^ aimed to compare long-term low-dose DOAC therapy (apixaban 2.5 mg BID) to a standard dual antiplatelet therapy scheme. The study was terminated after a planned interim analysis showed a significant reduction of bleedings and DRT at 3 months post-implant in the low-dose DOAC arm.^249^ The larger ongoing randomized ANDES trial^250^ may confirm these preliminary findings.

Future randomized studies should better define which antiplatelet and anti-thrombotic regimens are indicated after LAAC implant, in terms of safety and net outcomes, specifically focusing on patients who have contraindications to long-term therapy with OAC.

## Post-discharge left atrial appendage closure patient follow-up

In clinical studies, assessment of the patient's clinical status as well as of the anti-thrombotic medication was performed 6 months after the implant. In clinical routine, this is less common. Depending on the anti-thrombotic treatment regimen, however, it may be appropriate to schedule a counselling appointment.

One year after LAAC, the large majority of patients reduce the anti-thrombotic regimen to a single agent or stop this therapy. In controlled clinical studies TOE imaging was mandatory at the 12-month follow-up visit, although this is rarely done in clinical practice. It was noted, that depending on the device type and the medication used, not uncommonly DRT may occur late after implantation.^252^ This may be associated with an increased risk for stroke during long-term follow-up.^253^

Similarly, the presence of PDL at the 12-month imaging contributes to an increased rate of stroke.^254,255^ Both scenarios, DRT as well as PDL, have an impact on the future medical management of the patient. Therefore, it may be advisable to incorporate routine imaging at the 12-month follow-up visit into clinical routine but it is not a common practice in many centres.

In clinical studies with long-term follow-up, patient management beyond 1 year was usually limited to routine clinical assessment. Depending on comorbidities, it seems appropriate to tailor the individual follow-up schedule to the individual risk profile depending on co-existing medical conditions (e.g. every 6–12 months). Specific device-related imaging is not recommended.

In case of adverse clinical events such as stroke, unscheduled visits including imaging for DRT or PDL should be considered. Post- procedural risk, medication and follow-up are listed in Box 4.

**Box 4:** After LAAC: post-procedural risk, medication and follow-up

Same-day procedure or short hospitalization stay
TTE before discharge: device position and screening for pericardial effusion
Cardiac CT or TOE: 45 days to 3 months; screening for DRT and PDL
Device-related thrombosis (DRT): 0.23–2.2%
Peri-device leak (PDL): <3 mm: 12.9–27%; 3–5 mm: 3.7–9%; >5 mm: 0.4–1%
Post-procedural medication to reduce risk of DRT: DAPT or OAC 1–3 months, SAPT 6–12 months, reduced-dose DOAC 3–12 months (depending on risk for DRT and bleeding)
Endocarditis prophylaxis 6 months

## Other cardiac procedures after left atrial appendage closure

### Direct current cardioversion

Direct current cardioversion (DCCV) is frequently used in AF patients as part of a rhythm-control strategy. According to current guidelines, patients should be treated by anticoagulation at least 3 weeks before DCCV (AF duration >48 h) and 4 weeks after to prevent thromboembolic complications. However, patients after LAAC are often at high bleeding risk and therefore unsuitable for anticoagulation before and after DCCV. In two prospectively enrolled patient cohorts with a total of 242 LAAC patients, DCCV was used effectively without thromboembolic events despite the majority of patients being without anticoagulation before and after DCCV.^256,257^ In those studies, the majority of patients underwent TOE before DCCV to rule out device-related thrombus (DRT), large peri-device leaks, device malposition, and other cardiac thrombi.

Currently, the recommendations below can be used as a guide for DCCV in this patient group. There are no specific precautions for pharmacological cardioversion in LAAC patients.

- DCCV should be avoided the first 3 weeks after LAAC unless there is an acute indication, e.g. acute cardiac decompensation considered to be related to AF.
- TOE should always be performed before to rule out DRT, large PDL, device malposition, other cardiac thrombi. Computed tomography can be used as an alternative to TOE.
- Direct current cardioversion can be performed without anticoagulation before and after.
- Anticoagulation can be considered before and after in patients with a predicted very high risk of thromboembolic events (severe left atrial dilatation, pronounced spontaneous contrast or sludge in the left atrium, left ventricular ejection fraction < 25%, high CHA_2_DS_2_-VASc score, etc.) depending on an individual assessment of bleeding risk. Recent ACC/AHA/ACCP/HRS guidelines recommend (CoR: IIb, LOE: N-BR) pre-cardioversion imaging for LAAO patients who are not anticoagulated, and anticoagulation peri-cardioversion if there is a device-related thrombus or peri-device leak.^56^

### Atrial fibrillation catheter ablation

Atrial fibrillation catheter ablation and all other types of transcatheter cardiac ablation using various energy delivery sources (radiofrequncy (RF), cryotherapy, or pulsed-field) can be performed in patients after LAAC. Transoesophageal echocardiogram should be performed before AF ablation to rule out DRT and elective ablation should not be performed before the first follow-up imaging after LAAC which is typically done after 45 days or later. Anticoagulation post-ablation is recommended but adjusted according to the predicted bleeding risk for the individual patient.

### Transcatheter mitral interventions, transcatheter aortic valve replacement, and percutaneous coronary intervention

Transcatheter mitral interventions, TAVI, and PCI can all be performed in LAAC patients. Elective mitral intervention or TAVI should be planned not earlier than 45 days after LAAC or later, if possible. Transoesophageal echocardiogram should be performed before mitral intervention to rule out DRT or malposition of the device. For PCI, there are no specific LAAC-related precautions.

## Summary

The summary points for this practical guide are displayed in an unusual format. Those physicians who are considering referring a patient for an LAAC will often be asked by the patient a series of questions about the procedure, the necessary preparation and follow-up. The basis for answering these common questions has formed the content of this practical guide and the rationale and evidence base for the answers have been fully described in the guide for the benefit of the physician. The document is now summarized by proposing brief and accurate responses, in lay language, to these important questions.

### What is the left atrial appendage and why do we need to close it?

The LAA is a 2–6 cm-long, blind-ended, finger-like extension of the left atrium of the heart. It is a remnant of the development of the heart and does not have a significant role in the body. It is the place where most clots form in patients with AF, and if they detach these clots can cause a stroke.

### Am I a candidate for left atrial appendage closure?

Left atrial appendage closure is offered to patients who have AF, are at high risk for stroke, and cannot take oral anticoagulants (OACs—also known as blood thinners) for a prolonged period. The main reason for recommending the LAAC is because of serious bleeding complications of OACs. Also, LAAC may be offered to patients who had a stroke whilst they were optimally treated with OAC.

### How is left atrial appendage closure done?

The LAAC device is introduced into the heart using a catheter (long and thin tube) inserted through the veins in the groin. The collapsed device is expanded when it emerges from the tube when in the correct place within the heart to block the entrance to the left atrial appendage.

### Does it work?

According to the current information, for those patients able to take blood thinners (anticoagulants), LAAC may be equally effective to OAC drug therapy for stroke prevention, but does not cause long-term bleeding complications.

### Is it safe?

Yes. There is a small immediate risk related to the procedure. However, in experienced hands, this is considered a safe procedure, similar to other routine catheterization procedures.

### How about the long-term safety?

Late complications are very rare. The most common is DRT (clotting on the LAAC device) which is typically treated with a short period of OAC therapy.

### Is left atrial appendage closure a lifelong solution?

Yes. A device will achieve lifelong closure of the LAA. Over months, the surface of the device will be covered by the patient’s own tissue forming a smooth layer in continuation with the inner surface of the heart. This greatly reduces the likelihood of blood clotting on the device.

### Is there enough scientific evidence?

A few randomized clinical trials and many large registries have shown positive results. Larger clinical trials comparing the device to other medicines in a wider variety of patients are currently underway.

### Do I need to have any pre-procedural exams?

Often, a transoesophageal echocardiogram (TOE) or a cardiac CT (X-ray) is required before the procedure.

### Is atrial fibrillation going to stop after left atrial appendage closure?

No. LAAC is a stroke prevention therapy and does not cure AF.

### Do I need to be hospitalized for the procedure?

In most centres, the patient needs to stay overnight but same-day discharge is sometimes offered.

### Do I have to undergo general anaesthesia?

General anaesthesia is commonly used but some centres perform the procedure under light sedation or local anaesthesia.

### Is the procedure painful?

The procedure is not painful. It is performed through catheters, with a 4–5 mm incision of the skin in the groin. Pain after the procedure is unlikely, but a few days of avoiding vigorous activities is recommended to allow this small incision to heal.

### Will I stop taking blood thinners?

Yes. A few weeks after LAAC, the majority of patients may stop blood thinners. However, a short period of low-dose aspirin and/or clopidogrel therapy is required for some weeks, until the closure device is covered with the patient's own body tissue and healed. If you also have a reason other than AF for taking the OAC or antiplatelet therapy, you may have to continue the treatment.

### Do I need to have any exams after the procedure?

Yes. A TOE or CT is required, usually 6 weeks to 4 months after the procedure to check that everything is satisfactory.

### Can I feel the device in my chest?

There have been no reports of discomfort due to the device, nor any need for device removal for this reason.

### Can I have a magnetic resonance exam if needed in the future? How about airport security?

Yes. LAAC devices are compatible with up to 3T (strength of scanner) MRI scanners. Also, there are no special requirements for metal detectors at airport security checks.

### Do I need antibiotic treatment to prevent device infection?

During the implantation, a single dose of antibiotics is administered. After the procedure, antibiotic prophylaxis (for more invasive dental procedures, etc.) is recommended for a period of 6 months. After that antibiotics are not needed.

### Can I continue to play tennis, golf, and other sports after insertion of the device

Yes. You should avoid vigorous exercise for a few days after the procedure, but after that there is no reason to avoid sports or other vigorous activities. In fact, stopping OAC therapy reduces the risk of serious bleeding in case of any injury related to such activities.

### Is it possible for the device to dislodge?

This complication is very rare and it is manageable. A dislodgement after the healing phase is highly unlikely.

### Can the device be removed from the left atrial appendage?

The device becomes firmly attached to the tissue after it is inserted. The only way to remove it is by (minimally invasive) heart surgery, although this is rarely needed.

Please note that these Q&A's are written in order to help a referral physician to discuss with the patient being referred for placement of an LAAC device. Detailed explanations such as those that might be given by the implanting physician are not provided. The answers are not written primarily for the patient, although some words and phrases are chosen when they are more easily understood by the patient.

## Conclusions

The advice provided is fully in line with current guidelines and guidance documents provided by professional societies such as the European Society of Cardiology.

Research investigating the value of LAAC in comparison to approved alternatives is being rapidly conducted. For patients with high AF-related stroke risk who cannot be treated with anticoagulants to prevent stroke and other systemic emboli, LAAC is the only option and is often considered in such circumstances. These patients include those with anticoagulant-related major or life-threatening bleeding, a substantial threat of such bleeding in the presence of anticoagulants, failure of anticoagulants to prevent an embolic ischaemic stroke, or inability to comply sufficiently with anticoagulation treatment regimens, etc.

Left atrial appendage closure has been shown to be almost as effective and safer than VKA therapy but data comparing DOACs and LAAC are still insufficient to justify considering LAAC as a valid alternative to DOAC for treatment unless anticoagulation is contraindicated. For the time being, LAAC is a second-line therapy. However, many patients may qualify for LAAC treatment. These patients are spread throughout the full range of clinical specialties and care settings. For that reason this practical guide is offerred to physicians who are considering the referral of patients for LAAC therapy.
