# Correction to: Practical guide on left atrial appendage closure for the non-implanting physician: an international consensus paper

**DOI:** 10.1093/europace/euae142

**Published:** 2024-05-28

This is a correction to: Tatjana Potpara, Marek Grygier, Karl Georg Häusler, Jens Erik Nielsen-Kudsk, Sergio Berti, Simonetta Genovesi, Eloi Marijon, Serge Boveda, Apostolos Tzikas, Giuseppe Boriani, Lucas V A Boersma, Claudio Tondo, Tom De Potter, Gregory Y H Lip, Renate B Schnabel, Rupert Bauersachs, Marco Senzolo, Carlo Basile, Stefano Bianchi, Pavel Osmancik, Boris Schmidt, Ulf Landmesser, Wolfram Doehner, Gerhard Hindricks, Jan Kovac, A John Camm, Practical guide on left atrial appendage closure for the non-implanting physician: an international consensus paper, *EP Europace*, Volume 26, Issue 4, April 2024, euae035, https://doi.org/10.1093/europace/euae035

The originally published version of this article has been updated to correct the name of author Wolfram Doehner.

